# Plant growth promotion of the forage plant *Lupinus albus* Var. Orden Dorado using *Pseudomonas agronomica* sp. nov. and *Bacillus pretiosus* sp. nov. added over a valorized agricultural biowaste

**DOI:** 10.3389/fmicb.2022.1046201

**Published:** 2023-01-26

**Authors:** Marina Robas Mora, Vanesa M. Fernández Pastrana, Laura Luna Gutiérrez Oliva, Agustín Probanza Lobo, Pedro A. Jiménez Gómez

**Affiliations:** ^1^Department of Pharmaceutical Science and Health, Montepríncipe Campus, CEU San Pablo University, Madrid, Spain; ^2^Medicine Faculty, Montepríncipe Campus, CEU San Pablo University, Madrid, Spain

**Keywords:** *Bacillus pretiosus*, biofertilizer, organic fertilizer, PGPB, *Pseudomonas agronomica*, rhizobacteria, plant residue valorization

## Abstract

**Introduction:**

The overexploitation of natural ecosystems and the evolution of climate change currently force us to design new strategies for more sustainable agronomic uses. The recovery of plant residues, as an alternative to agrochemicals, can help alleviate these problems, for example, through its use for the synthesis of biofertilizers. In this work, the effect of the organic fertilizer matrix ORGAON^®^ from the valorization of horticultural waste is tested, to which two strains of bacteria (and their consortium) are added (SAICEU11^T^ identified as *Bacillus pretiosus* and SAICEU22^T^ identified as *Pseudomonas agronomica*), selected for their demonstrated ability to promote plant growth (PGPB), on the lupine forage plant (*Lupinus albus*).

**Methods:**

For the synthesis of the biofertilizer, both strains were added to the ORGAON^®^ organic matrix separately, until reaching a final optical density (OD) of 0.5 McFarland in each case in the irrigation matrix. As a control, sterile ORGAON^®^ (ORGAON^®^st) was used, also supplemented with the PGPB strains and a chemical fertilizer widely used in agronomy (Chem-F). With these treatments, a 6-week experiment was started under controlled laboratory conditions and on agricultural substrate, to recreate field conditions as accurately as possible. All the tests were carried out with 9 repetitions and 3 replicates of each treatment. After harvest, the improvements on the following biometric variables were studied for each treatment: total weight (Weight_T, g), shoot weight (Weight_S, g), root weight (Weight_R, g), number of leaves (Leaves, No.), shoot length (Length_S), root length (Length_R) and number of secondary roots (Roots, No.). Likewise, the identification of the tested strains and their description as new species was carried out. For this, they were studied from the phenotypic point of view (Transmission electron microscopy (TEM), metabolic profile, PGP activities, fatty acid profile and Matrix-assisted laser desorption/ionization time-of-flight (MALDI-TOF)) and genotypic (sequencing of the main housekeeping genes and sequencing of the whole genome, genomic characteristics (dDDH and ANI) and phylogenetic analysis).

**Results and discussion:**

After the statistical analysis of the results, it is shown that the individual addition of both strains on the ORGAON^®^ and ORGAON^®^st organic matrix improve certain biometric variables. In the case of the SAICEU11T (Bacillus pretiosus) strain, the variables root weight (Weight_R, g), total weight (Weight_T, g) and length of the plant, and number of secondary roots (Roots, No.) significantly improve, while in the case of the strain SAICEU22^T^ (*Pseudmonas agronomica*), a significant improvement of root length (Length_R) and number of secondary roots (Roots, No.) is demonstrated. On the other hand, the genotaxonomic analysis showed that both species have not been described to date. The identification based on the main housekeeping genes, show that for the *Bacillus* strain (SAICEU11^T^) the sequence similarity of the *16S rRNA* was 100%, *gyrB* 92.69%, *rpoB* 97.70% and rpoD 94.67%. For the Pseudomonas strain (SAICEU22^T^) the results were 100% for 16S rRNA, 98.43% for rpoD and 96.94% for *gyrB*. However, in both cases, the dDDH and ANI values, as well as the phylogenetic analysis, show that both species are below the species threshold, which would support the hypothesis that both are new species, in line with the chemotaxonomic results obtained by MALDI-TOF spectrometry and fatty acid profile. To verify the biosafety in their handling and release into the natural environment, we have ruled out the presence of genes that encode virulence factors or resistance to antibiotics, concluding that they are suitable for use in the field to improve the yield of crop plants. Type strains are SAICEU11T (= DSM 114702^T^ = CECT30674^T^) for Bacillus pretiosus and SAICEU22^T^ (= DSM 114959^T^ = CECT30673^T^) for *Pseudomonas agronomicae*.

## 1. Introduction

The term “land resources” includes the physical, biotic, environmental, infrastructure and socio-economic components of a natural land unit, which includes the resource of surface fresh water, and close to the surface, essential for all human activity, including agricultural uses. Thus, the interaction between the components of environmental resources is vital for the determination of the productivity and sustainability of agroecosystems (Dubois, 2011). Unfortunately, the resilience of the system to resist and adapt to natural (such as climatology, availability of water resources) and human-induced (pollution, overexploitation, land uses and management) changes and fluctuations it fails to alleviate fatigue and impoverishment of soil and other natural resources. Thus, abiotic stress can cause the components of an ecosystem, such as the proportion of nutrients or microorganisms that live in it, to be affected (Fiodor et al., 2021). In this sense, the increase in the demand for food induces the use of a greater number of chemical fertilizers, which increase prices, overexploit resources, increase the pollution of natural environments and exacerbate the problem of soil impoverishment (Kumar et al., 2021). For these reasons, there is a scientific-technical interest in developing biotechnological tools that can contribute to alleviating this scarcity and supplies without impoverishing soils or negatively affecting ecosystems.

The recovery of agricultural plant residues to generate liquid organic fertilizers can offer an alternative to the abuse of chemical fertilizers (Witzgall et al., 2021). However, the complexity of the biomolecules that constitute them requires the participation of microorganisms for their transformation into simpler molecules, facilitating their absorption by plants. In this sense, the addition of plant growth-promoting bacteria (PGPB) strains can indirectly favor the transformation of organic matter favoring its root absorption (Katsenios et al., 2022). Likewise, its direct action in stimulating plant growth can effectively contribute to the improvement of agricultural production. Both effects seek to optimize the agronomic use of soils, reduce the demand for water and its corresponding abiotic pressure on aquifers, thus avoiding their contamination and minimizing their overexploitation (Lara Mantilla et al., 2011; Hakim et al., 2021).

Numerous potentially PGPB species are known in the *Bacillus* and *Pseudomonas* genera, especially for the great diversity of species that both possess. *Bacillus* genus was first described by Smith (1957). In turn, the *Pseudomonas* genus was first described by Migula (1894). The classification of both genera is detailed in the 2nd Edition of Bergey’s Manual of Systematic Bacteriology (Imhoff et al., 2005). The phylogeny of both genera is not entirely clear (Patel and Gupta, 2020; Lalucat et al., 2021) and its taxonomic ordering is being debated (La Duc et al., 2004; Torres Manno et al., 2020; Lalucat et al., 2021). The use of the housekeeping *16S rRNA* gene for the identification of strains is not enough, given that strains with very high homology percentages are known with respect to species with which they can differ greatly in their phenotype and even gene content (Gomila et al., 2015; Dunlap, 2019). For this reason, in order to obtain a more robust phylogeny, analyses of a greater number of additional housekeeping genes have recently been incorporated, among which are *gyrB*, *rpoB* and *rpoD* in the case of the genus *Bacillus* (Yamada et al., 1999; La Duc et al., 2004), and *gyrB* and *rpoD* for the genus *Pseudomonas* (Yamada et al., 1999). However, the rise of Next Generation Sequencing (NGS), the precision and speed with which complete genomes are obtained, makes it necessary to provide more information to taxonomically classify species (Young and Gillung, 2020). The genomotaxonomy of new species includes the calculation of the whole genome DNA–DNA hybridization (dDDH) with evolutionarily close species, the average value of nucleotides (ANI) and the entire genome sequencing (Volpiano et al., 2021).

*Lupinus albus* is a fodder plant native to the Mediterranean area commonly used as food, due to its easy digestibility and high protein content (Hufnagel et al., 2020). Currently, the demand for vegetables with high protein content is increasing. A good example of this is lupine (*Lupinus albus*; Alkemade et al., 2021), an easy to grow plant with a root system that allows its growth in unfavorable environments and impoverished soils (Hufnagel et al., 2020). For these reasons, this species has been widely studied and is commonly used in biological tests. In the present work the effect of ORGAON^®^ is tested, an organic fertilizer whose raw material is the recovered plant residues originating from horticultural plantations. Likewise, the collaborative effect that the PGPB SAICEU11^T^ and SAICEU22^T^ included in the matrix of said fertilizer exert on *L. albus* plants is tested.

Based on the phenotypic and genotypic analysis SAICEU11^T^ and SAICEU22^T^ are proposed as new species belonging to the *Bacillus* and *Pseudomonas* genera, respectively. Its genomic analysis allows us to verify that both are harmless to both the environment and living beings. Due to its innocuousness and potential PGP, biological tests were carried out on *Lupinus albus* var. Dorado irrigated with a matrix of organic fertilizer ORGAON^®^ in comparison with a traditional chemical fertilizer, both supplemented with the PGPB strains.

## 2. Materials and methods

### 2.1. Isolates

#### 2.1.1. Permission to reuse and copyright

SAICEU11^T^ and SAICEU22^T^ strains were isolated from the *Medicago sativa* rhizosphere grown mercury contaminated soils of the Almadén Mining District (Ciudad Real, Spain; Robas et al., 2021).

#### 2.1.2. Plant growth promotion activities (PGP) characterization

To determine the *in vitro* production capacity of auxins (IAA), a colorimetric technique was used with the Van Urk Salkowski reagent, using the method described by Ehmann (1977). Bacteria were grown in LB medium (Texas, United States) at 28°C for 4 days without agitation and in darkness. The liquid medium was centrifuged after incubation, obtaining the supernatant. Then, 1 ml of it was mixed with 2 ml of Van Urk Salkowski reagent (2% FeCl_3_ in 35% of HClO_4_ solution) and kept in darkness. Optical density (OD) was measured at 530 nm after 30 min and 120 min. The results were quantified in ppm (g mL^−1^). Glick (1995) protocol was followed to differentiate the degradation of 1-aminocyclopropane-1-carboxylic acid (ACC) by the action of the enzyme ACC deaminase (ACCd) from bacteria having the ability to fix nitrogen. The culture medium contained 1.8% Bacto-Agar (Difco Laboratories, Detroit, United States), which has low nitrogen content, supplemented with ACC (30 mmol L^−1^). The Petri plates (90 × 14 mm) were then inoculated and cultured for 3 days at 28°C, checking the growth daily. The results were evaluated qualitatively (presence/absence of ACCd enzyme). Siderophore production was quantified using the Chrome Azurol S (CAS) agar, described by Alexander and Zuberer (1991). The interpretation was based on the quantitative analysis of the production of siderophores, manifested by the appearance of a halo around the bacterial colonies after 72 h of incubation at 28°C. The ability to solubilize phosphates was tested following the protocol described by De Freitas et al. (1997). Tricalcium-phosphate agar medium (TPM) (Nautiyal, 1999) was used, with a final pH adjusted to 7 with 1 mole L^−1^ HCl. After inoculation, plates were incubated at 28°C for 72 h. The inorganic phosphates solubilizer colonies showed distinct clarification halos that were evaluated qualitatively (presence/ absence). All PGPB activities were analyzed in triplicate.

### 2.2. Biological assays for promoting plant growth with ORGAON^®^ biofertigation; an agriculture residue

#### 2.2.1. General cultivation conditions of the two strains

The strains were obtained from the rhizosphere of Medicago sativa, from soils contaminated with mercury (Hg) from the mining district of Almadén (Ciudad Real, Spain) following the protocol described in Robas et al. (2021). Briefly, for their isolation, an extract of the rhizospheric soil was made and they were classified as mercuritolerant following the criteria established by Mathema et al. (2011). To measure its maximum bactericidal concentration (MBC) to Hg, the agar medium for standard methods (SMA, Pronadisa^®^, Madrid, Spain) supplemented with different concentrations of HgCl_2_ (400, 350, 200, 175, 150, 100, 87.5, 75, 50, 43.75, and 25 μg mL^−1^).

#### 2.2.2. ORGAON^®^ As recovered waste

ORGAON^®^ is an organic fertilizer that comes from plant waste recovered by the company Biaqui S.L. (Almería, Spain). The agricultural plant remains are pressed and the liquid fraction constitutes a valuable extract that, after its transformation, is recovered as organic plant-based fertilizer. It is, therefore, a very valuable raw material, with a high content of complex organic compounds that must be transformed into inorganic forms for plant assimilation. Its enrichment with the SAICEU11^T^ and SAICEU22^T^ PGPB individually allows the elaboration of an organic biofertilizer, environmentally friendly. The physicochemical characteristics of ORGAON^®^ are described in Robas Mora et al. (2022).

To carry out the biological assay, ORGAON^®^ fertilizer was also tested sterilized (ORGAON^®^st) in parallel, by exposing a liquid sheet (2 mm) to UV light (265 nm) for 30 min.

For producing the biofertilizer, the inoculum of the SAICEU11^T^ strain and SAICEU22^T^ strain (optical density (OD) 0.5 McFarland) were added on this matrix, separately. To do this, 100 ml of a 5.0 McFarland suspension of SAICEU11^T^ and SAICEU22^T^ were added to 1 l of the 1/500 ORGAON^®^/ORGAON^®^st dilution, in each case and separately (Robas Mora et al., 2022). The pure cultures of the SAICEU11^T^ and SAICEU22^T^ strains were kept in cryo pearls at −80°C, prior to their use. They were recovered in SMA (Pronadisa^®^, Madrid, Spain), incubation 24 at 37°C, coinciding with the stationary phase of the culture. Subsequently, it was verified by Gram stain that the cultures were maintained in pure culture.

#### 2.2.3. Biological assay

Pre-germinated *Lupinus albus* var. Dorado seeds, supplied by the Institute of Agricultural Research Finca La Orden-Valdesequera (Badajoz, Spain), were used. Prior to the growth assay, seeds were superficially sterilized by a 30 s ethanol washing (70% *v*/*v*). After that time, it was washed with plenty of sterile mineral water. Pre-germination was carried out on sterilized vermiculite substrate, at field capacity (at saturation, moment prior to the drainage of water), in PVC trays that were kept in darkness and stable room temperature (20°C ± 2°C) for 96 h until the appearance of a visible emerged radicle of 2.0 ± 0.2 cm. The agricultural soil was obtained from the plot where field trials are currently being carried out, located in Villanueva de San Mancio (GPS coordinates 21°92′N 5°03′O, Valladolid, Spain). It was sieved (0.5 cm) and the resulting product was mixed with sterile river sand 50/50 (*w*/*w*) to standardize granulometry in all treatments and replicates. Seedbeds of 10 cm × 8 cm were used on trays of 40 cm × 35 cm (three replicates per treatment and per tray) each with nine *Lupinus albus* pre-germinated seeds, per treatment (Table 1).

**Table 1:** Hg MBC: Hg maximum bactericidal concentration; IAA: 3-indoleacetic acid production; ACCd: 1-aminocyclopropane-1-carboxylic acid deaminase production; SID: siderophore production; SUN. PO_4_^3−^: phosphate solubilizing capacity.

ID	Hg MBC (μg ml^−1^)	IAA (μg ml^−1^)	ACCd (p/a)	SID (cm)	SOL. PO_4_^3−^ (p/a)
SAICEU11^T^	87,5	5,61	−	1	−
SAICEU22^T^	160	5,85	+	1	−

The irrigation matrix was ORGAON^®^ (organic fertilizer), at an optimal experimental concentration of 1/500 (V_ORGAON_/V_H2O_). Aditionally, ORGAON^®^st fertilizer was tested. The tests were carried out under controlled laboratory conditions (18°C ± 3°C, natural light contribution and relative humidity of 30%) and two weekly irrigations (experimental average volumes of 50 ml) with the biofertilizer (ORGAON^®^/ORGAON^®^st, supplemented with the SAICEU11^T^ strain and ORGAON^®^/ORGAON^®^st, supplemented with the SAICEU22^T^ strain). Controls were done with a traditional chemical fertilizer (Chem-F) or the organic fertilizer ORGAON^®^/ORGAON^®^st, in all cases without supplementation with the SAICEU11^T^ strain or SAICEU22^T^ strain. Chem-F consisted in a fertilizer low in chloride with the following formulation: (K2HPO4; 5%); (K 2SO4; 14%); (MgSO₄; 2.5%).

For the dilutions of the ORGAON^®^ and the chemical fertilizer Chem_F, mineral water was used, with the composition detailed in Table 2.

**Table 2:** Mineral water chemical composition.

Components	Formula	Content (mg L^−1^)	Content (mmol L^−1^)
Bicarbonate	HCO_3_^−^	222.2	3.641
Chloride	Cl^−^	9.3	0.262
Sulphate	SO_4_^2−^	56.5	0.588
Calcium	Ca^2+^	71.3	1.779
Magnesium	Mg^2+^	10.9	0.448
Potassium	K^+^	4.4	0.113
Sodium	Na^+^	15.9	0.692
Silica	SiO_2_	33.3	0.554
Dry residue	–	314	–

The harvest was carried out after 6 weeks and involved the extraction of the shoot and root parts of each plant. The root part was washed with distilled water to remove all traces of substrate.

Nine pregerminated seeds (repetitions) were used for each treatment. Each fertigation treatment was tested in triplicate (replicates): ORGAON^®^, ORGAON^®^st and Chem_F added with SAICEU11^T^, SAICEU22^T^, the consortium of both or the control without addition, making a total of 27 pots per strain/ consortium, according to the experimental design shown in Table 3.

**Table 3:** Experimental design.

	ORGAON^®^	ORGAON^®^st	Chem_F	Total
CONTROL	9 seeds × 3 replica	9 seeds × 3 replica	9 seeds × 3 replica	27 pots
SAICEU11^T^	9 seeds × 3 replica	9 seeds × 3 replica	9 seeds × 3 replica	27 pots
SAICEU22^T^	9 seeds × 3 replica	9 seeds × 3 replica	9 seeds × 3 replica	27 pots
CS	9 seeds × 3 replica	9 seeds × 3 replica	9 seeds × 3 replica	27 pots

The following biometric parameters were analyzed: total weight (Weight_T, g), shoot weight (Weight_S, g), root weight (Weight_R, g), number of leaves (Leaves, N_o_.), shoot length (Length_S), root length (Length_R) and number of secondary roots (Roots, N_o_.). The two bacteria isolates were subjected to mutual compatibility test by cross streak method in SMA (Pronadisa^®^, Madrid, Spain).

#### 2.2.4. Statistical analysis

To evaluate the effects of treatment on all biometric variables, for each irrigation matrix, an analysis of variance (one-way ANOVA) was performed. When significant differences appeared (value of *p* < 0.05), a Duncan *post-hoc* analysis was performed to identify those fertigation treatments that explain the difference between bacterial treatments. The SPSS v.27.0 program (IBM Corp, Armonk, NY, United States) was used.

### 2.3. New species/strains characterization and description

#### 2.3.1. Transmission electron microscopy (TEM)

To determine the size and shape of the analyzed strains, the Prism E Scanning Electron Microscope (SEM) (Thermo Fisher Scientific Inc., Walthman, USA) was used. The culture was observed in suspension. A drop of Formvar on 200 mesh Cu TEM grids was used as a sample holder in the grid for TEM microscopy. The measurement conditions were: 10 mm working distance; 24 pA electron current; electron acceleration of 30 kV and 2-point size, pressure of 375 Pa, at 4°C and a humidity of 50%. The electron microscopy images were obtained by the research support service (SAI) of “X-ray diffraction and scanning electron microscopy” (SAI-DRX-MEB) of the CEU-San Pablo University (Madrid, Spain).

#### 2.3.2. Biochemical tests

Oxi/Ferm Pluri Test^®^ (Liofilchem, Italy) was used. The automatic characterization was then carried out using the VITEK^®^-2 GN identification cards (bioMérieux, Marcy-l’Étoile, France). The motility of the bacterium was tested in Motility Test Agar (Liofilchem, Italy). Antimicrobial sensitivity was determined using E-test in Müller Hinton agar (Pronadisa^®^ Madrid, Spain) using different antibiotics for each strain. For SAICEU11^T^ strain we used cefepime and sulfamethoxazole and trimethoprim (bioMérieux, Marcy-l’Étoile, France); amoxicillin, amoxicillin-clavulanic acid, cefotaxime, cefpirome ciprofloxacin and nalidixic acid (Liofilchem, Italy). For SAICEU22^T^ strain we used piperacillin and piperacillin with tazobactam, cefepime (bioMérieux, Marcy-l’Étoile, France); ceftazidime, imipenem, imipenem with EDTA, amikacin, gentamicin, and ciprofloxacin (Liofilchem, Italy).

Mercury MBC was performed in Müller Hinton agar (Pronadisa^®^, Madrid, Spain), supplemented with different concentrations of HgCl_2_: 400, 350, 200, 175, 150, 100, 87.5, 75, 50, 43.75, and 25 μg mL^−1^. MBC was determined as the lowest concentration of HgCl_2_ capable of inhibiting > 99.9% of bacterial growth.

#### 2.3.3. Fatty acids profile

Fatty acids spectrum was determined following the protocol recommended by MIDI Microbial Identification System (Sasser, 2001) at Spanish Collection of Type Cultures (CECT, Valencia, Spain). An Agilent 6,850 gas chromatograph was used, with the MIDI Microbial Identification System using the TSBA6 method (MIDI, 1999). For this, Trypticase soy broth (TSBA, Condalab^®^, Torrejón de Ardoz, Madrid) as culture medium was used, and an incubation temperature of 28°C for 24 h.

#### 2.3.4. Matrix-assisted laser desorption/ionization time-of-flight (MALDI-TOF)

Matrix-Assisted Laser Desorption/Ionization Time-Of-Flight (MALDI-TOF) was used. It was carried out in the Vitek^®^ MS IND system (BioMérieux, Marcy-l’Étoile, France) at the Carlos III Health Institute in Madrid. The slides were inoculated with a sterile loop. 1 l of the matrix solution (Vitek^®^ MS-CHCA: mixture of 3.10 g of 2.5-dihydroxy 36 benzoic acid dissolved in 100 ml of water-ethanol-acetonitrile in 1/1/1 ratio) was added to each well and left to dry at room temperature. The mass spectra were generated with the Axima Assurance system (Shimadzu Corporation, Kyoto, Japan), using the Shimadzu Launchpad software program and the SARAMIS MS-ID v1 database application (AnagnosTee GmbH) for automatic measurement and identification. All strains were analyzed in duplicate. No pre-treatment was used before their inoculation on the slide. High confidence identification was considered when the assessment was equal to or greater than 97%.

#### 2.3.5. Sequencing analysis

For genomic DNA extraction, QIAamp DNA Kit (QIAGEN^®^, Hilden, Germany) was used. The entire genome was sequenced in the genomics at Carlos III Health Institute (Madrid, Spain). With the obtained data, the *de novo* assembly and gene annotation was performed, corresponding to the *Bacillus* and *Pseudomonas* genus. Sequencing was carried out with the Illumina^®^ platform in paired end format (2 × 300; Illumina^®^, Inc., San Diego, CA, United States). The quality control analysis was performed with the FastQC program (Andrews, 2010). Adapters were removed from FASTQ files using the CUTADAPT tool (Martin, 2011). The filtering was carried out with the Prinseq platform (Schmieder and Edwards, 2011). In the filtering, in addition to quality, a minimum size of 100 bp was used and those sequences with the presence of more than 5% of Ns were rejected. Neither the first 15 nucleotides nor the last 15 were considered due to low signal quality. The FASTQCollapser tool was used to remove duplicate readings and FASTQIntersect to remove sequences that were not in both readings (forward and reverse) of the file. For the sample *de novo* assembly the SPAdes assembly software (Bankevich et al., 2012) was used to achieve the scaffolds reconstruction. The metrics of the assemblies were obtained through SeqEditor (Hafez et al., 2021). Genome hybridization and identity percentages of the *16S rRNA*, *gyrB*, *rpoB* and *rpoD* genes were analyzed using the basic local alignment search tool BLAST (Altschul et al., 1990).

The *16S rRNA* gene was analyzed using the NCBI local alignment tool (BLAST). The search was restricted to Sequences from type material. The SAICEU11^T^ and SAICEU22^T^ strains genomes were analyzed using the TYGS server (type strain genome server; Meier-Kolthoff and Göker, 2019) and they were used as a basis for the realization of the phylogenetic trees. For this, the MASH algorithm was used, which gives an approximation to the possible intergenic relationships. (Ondov et al., 2016). Also, with the JSpeciesWS ANI value. When the software identifies that the species differ in any of the analyzed criteria, it identifies it with different colors in section “species cluster.” After that, dDDH was also performed with TIGS server (Meier-Kolthoff and Göker, 2019).

Several clinically important antimicrobial resistance genes were searched through functional annotation data generated from Rast, Comprehensive Antibiotic Resistance Database (CARD), ARG-ANNOT V4 and ResFinder 4.1 annotation lines.

#### 2.3.6. Gene identification and annotation

The Uniprot platform (The Uniprot Consortium, 2021) and the NCBI database were used. For gene annotation, the Prokka program (*rapid prokaryotic genome annotation*) was used (Seemann, 2014). To check the presence of virulence genes and pathogenicity factors, the Virulence Factor Database (VDFB:) was used (Yang et al., 2007).

## 3. Results

### 3.1. Plant growth-promoting biological assays by biofertirigation

Biological tests were carried out to know the effect of biofertigation on the plant with two compound biofertilizers, one by the SAICEU11^T^ strain and the other by the SAICEU22^T^ strain added on ORGAON^®^ and ORGAON^®^st (sterile). The consortium composed of both strains was also tested, once it was verified with the compatibility test that both strains do not exert interspecific competition phenomena between them. Its efficacy is compared to the traditional chemical fertilizer (Chem_F). In Table 4 the value of ps of the ANOVA results are shown. What is highlighted are those biometric parameters that underwent some significant variation (value of *p* < 0.05) with each fertigation condition. Only for these biometric parameters that are dependent on the type of irrigation, a *post-hoc* analysis (Duncan’s test) is performed to find out which irrigation is the one that justifies such variations. Full results can be found at length in the Supplementary Tables 1A–D.

**Table 4:** Significance of the differences between values (value of *p*) after the ANOVA analysis, for each bacterial treatment and consortium.

	SAICEU11^T^	SAICEU22^T^	CS
Weight_T	**0.036**	0.185	0.397
Weight_S	0.085	0.220	0.289
Weight_R	**<0.001**	0.061	0.423
Leaves	0.160	0.600	0.328
Length_S	**0.032**	0.023	0.070
Length_R	0.066	**<0.001**	**0.049**
Roots	**0.003**	**0.000**	**0.004**

The differences for each biometric parameter are statistically significant when the value of *p* < 0.05 (bold).

**Figure 1:**
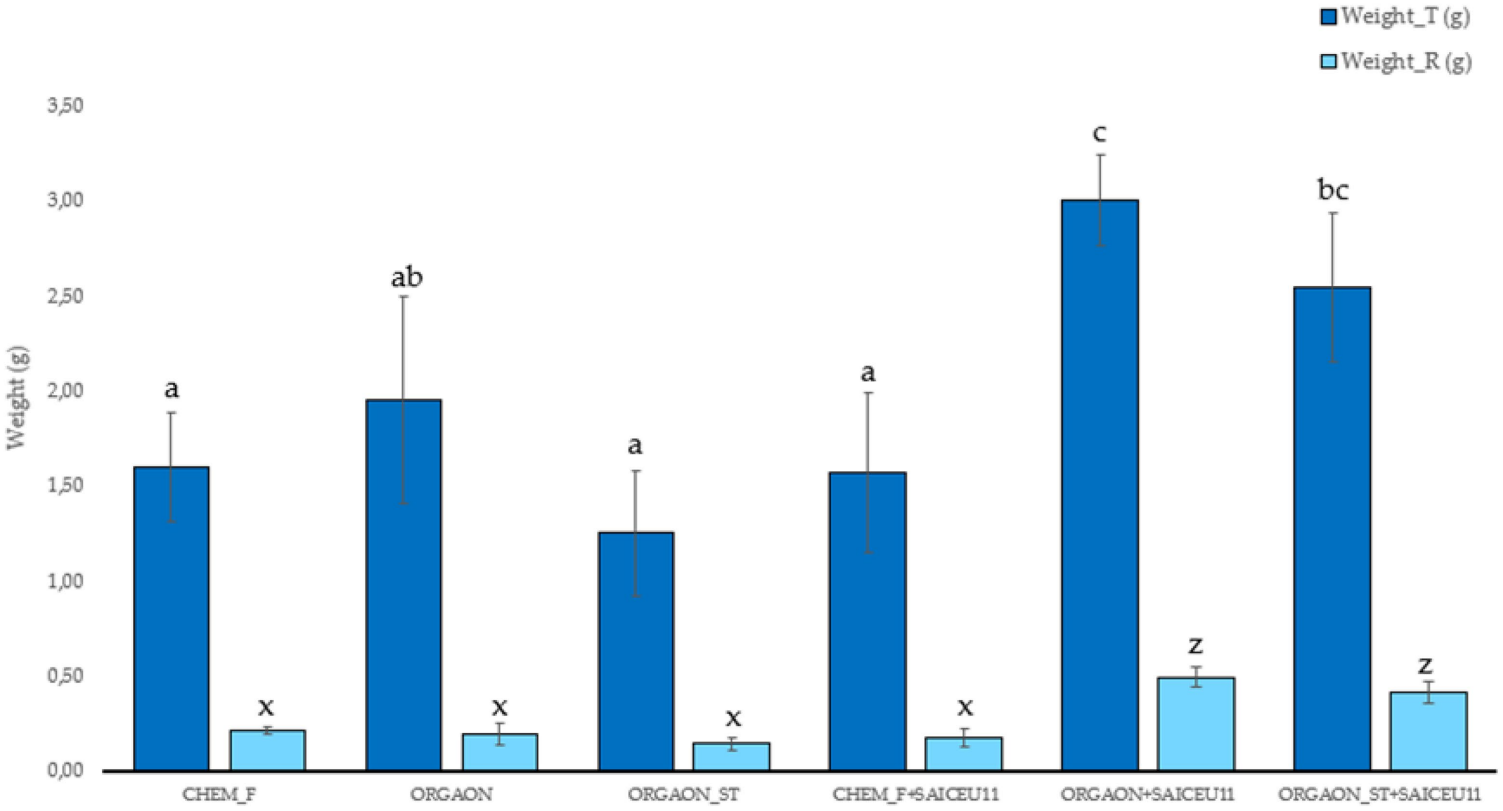
Weight biometrics of lupine plants subjected to different irrigation treatments with biofertilizer with SAICEU11^T^. Different letters denote statistically significant differences according to Duncan test (*p* < 0.05) for total plant weight (a, ab, and c) and root weight (*x* and *z*).

**Figure 2:**
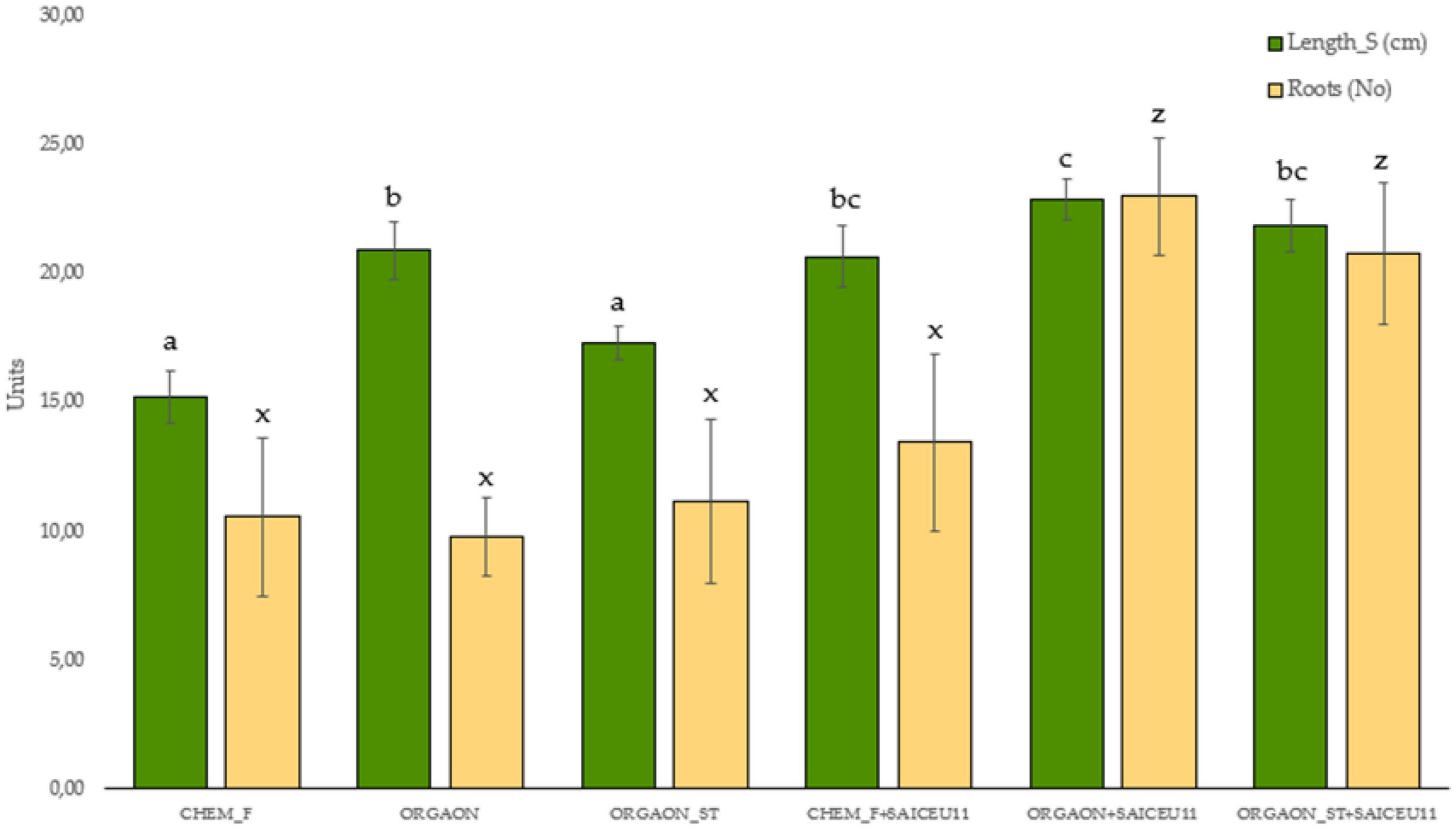
Biometrics of lupine plants subjected to different irrigation treatments with biofertilizer with SAICEU11^T^. Different letters denote statistically significant differences according to Duncan test (*p* < 0.05) for shoot length (a, b, bc, and c) and the number of secondary roots (*x* and *z*).

**Figure 3:**
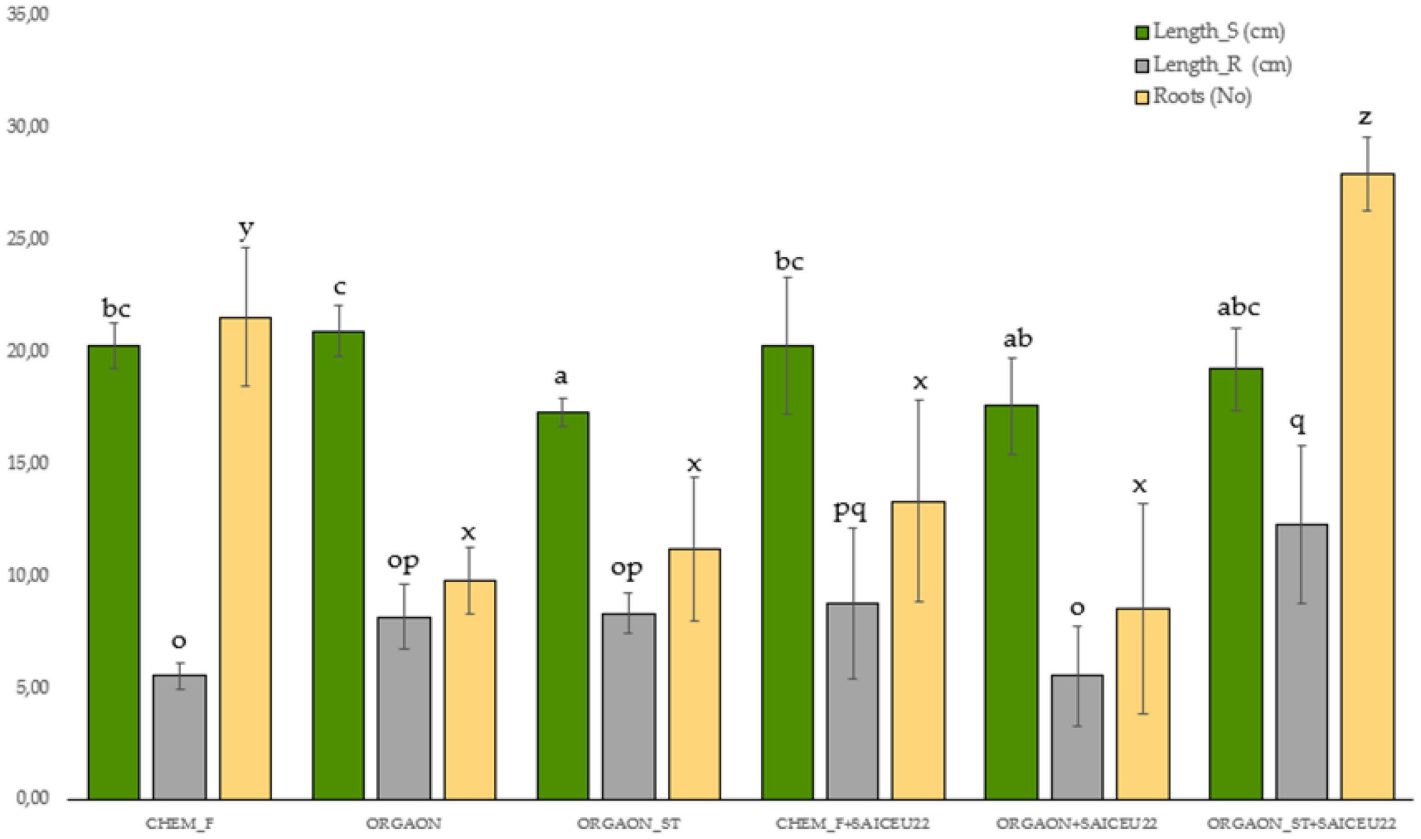
Weight biometrics of lupine plants subjected to different irrigation treatments with biofertilizer with SAICEU22^T^. Different letters denote statistically significant differences according to Duncan test (*p* < 0.05) for shoot length (a, ab, abc, bc, and c), the root length (o, op, pq, and q) and the number of secondary roots (*x*, *y*, and *z*).

**Figure 4:**
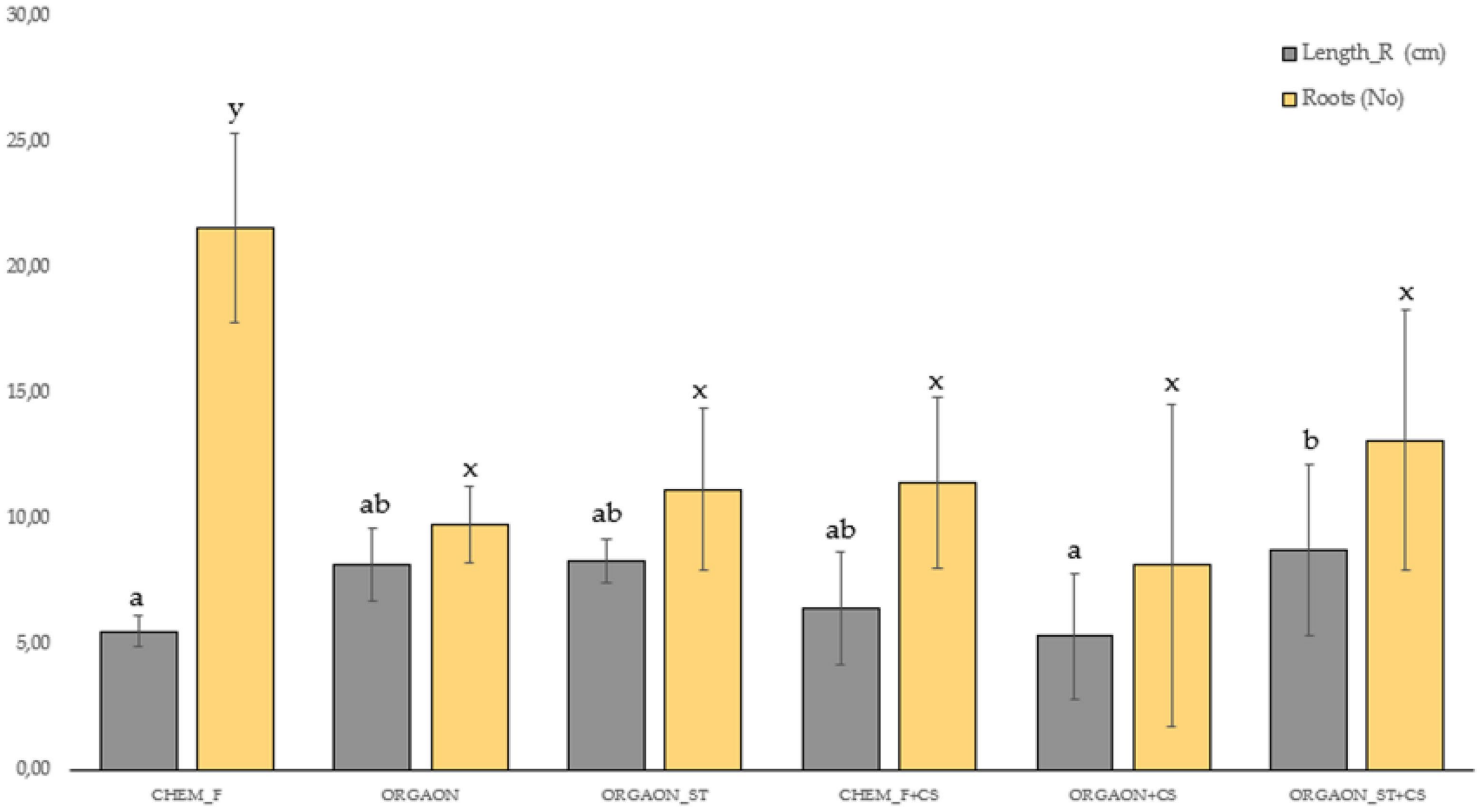
Weight biometrics of lupine plants subjected to different irrigation treatments with biofertilizer with both SAICEU11^T^ and SAICEU22^T^ (CS). Different letters denote statistically significant differences according to Duncan test (*p* < 0.05) for the root length (a, ab, and b) and the number of secondary roots (*x* and *y*).

Next, only those biometric parameters that are influenced by the type of fertigation are represented graphically. The *post-hoc* analysis carried out (Duncan’s test) allows the identification of those biometrical parameters that explain such differences (Figures 1–4).

After the *post-hoc* analysis with Duncan’s test, it was found that the treatments of ORGAON^®^ and ORGAON^®^st supplemented with strains SAICEU11^T^ and SAICEU22^T^ showed statistical differences in biometrics, with respect to the plants treated with Chem-F as with fertilizers in the absence of added strains.

The sterilization of the ORGAON^®^ fertilizer carried out to eliminate the native microbiota of the organic recovered residue, allowed to verify the promoting effect of the s strains SAICEU11^T^ and SAICEU22^T^ in the biometric variables (Figures 1–3).

In the compatibility test between SAICEU11^T^ and SAICEU22^T^ strains, no inhibition was observed on the cross point in any of the combinations, which indicate the compatibility among the isolates.

### 3.2. Species identification

The length of the *16S rRNA* gene of the SAICEU11^T^ strain was 1,359 bp; on the other hand, the length of this gene in the SAICEU22^T^ strain was 1,125 bp. The percentages of similarity of the *16S rRNA* gene greater than 98.65% were analyzed, calculating the dDDH and ANI values for each of them (Tables 5A,B).

**Table 5A:** Similarity of the *16S rRNA* gene greater than 98.65% of all type strains against SAICEU11^T^.

Description	Per Ident	ANIb	dDDH
*Bacillus toyonensis* BCT-7112*^T^*	100	90.68	43.8
*Bacillus wiedmannii* FSL W8-0169*^T^*	100	95.99	69.6
*Bacillus pacificus* EB422*^T^*	100	92.77	66.5
*Bacillus mobilis* 0711P9-1*^T^*	100	94.32	58.2
*Bacillus cereus* ATCC 14579^T^	99.33	90.93	43.9
*Bacillus fungorum* 17-SMS-01*^T^*	99.93	92.87	52.9
*Bacillus thuringiensis* ATCC 10792*^T^*	99.93	90.87	43.9
*Bacillus paramobilis* BML-BC017*^T^*	99.93	94.40	—
*Bacillus sanguinis* BML-BC004*^T^*	99.93	93.64	—
*Bacillus cereus* CCM 2010*^T^*	99.93	90.99	43.9
*Bacillus paranthracis* MCCC 1A00395*^T^*	99.92	92.63	50.9
*Bacillus proteolyticus* MCCC 1A00365*^T^*	99.92	89.31	40.6
*Bacillus hominis* BML-BC059*^T^*	99.85	89.27	—
*Bacillus anthracis Vollum* ATCC 14578*^T^*	99.85	92.65	50.5
*Bacillus albus* MCCC 1A02146*^T^*	99.85	93.05	54.9
*Bacillus paramycoides* MCCC 1A04098*^T^*	99.84	88.54	37.6
*Bacillus tropicus* MCCC 1A01406*^T^*	99.84	92.94	51.5
*Bacillus clarus*	99.79	84.92	—
*Bacillus mycoides* ATCC 6462*^T^*	99.71	89.64	40.1
*Bacillus pseudomycoides* DSM 12442*^T^*	99.56	81.56	27.1
*Bacillus gaemokensis* BL3-6*^T^*	99.44	81.24	26.5
*Bacillus bingmayongensis* FJAT-13831*^T^*	99.31	81.60	26.9
*Bacillus pacificus* EB422^T^	99.22	92.80	51.3

**Table 5B:** Similarity of the *16S rRNA* gene greater than 98.65% of all type strains against SAICEU22^T^.

Description	Per. ident	ANIb	dDDH
*Pseudomonas alvandae* SWRI17*^T^*	100.00	94.29	60.4
*Pseudomonas rhizophila* S211T*^T^*	99.91	85.58	46.3
*Pseudomonas canavaninivorans* HB002*^T^*	99.91	94.13	60.5
*Pseudomonas bijieensis* L22-9 *^T^*	99.73	86.67	34.5
*Pseudomonas brassicacearum subsp. neoaurantiaca* ATCC 49054 *^T^*	99.73	93.43	35.3
*Pseudomonas corrugata* NCPPB 2445 *^T^*	99.63	85.00	31.5
*Pseudomonas zarinae* SWRI108 *^T^*	99.56	87.03	35.2
*Pseudomonas fluorescens* F113 *^T^*	99.47	86.99	25.0
*Pseudomonas kilonensis* 520–20 *^T^*	99.46	87.04	35.3
*Pseudomonas thivervalensis* SBK26 *^T^*	99.46	87.28	35.4
*Pseudomonas mediterranea* DSM 16733 *^T^*	99.38	85.34	32.1
*Pseudomonas viciae* 11 K1 *^T^*	99.38	86.04	33.7
*Pseudomonas brassicacearum* DBK11 *^T^*	99.37	87.34	35.3
*Pseudomonas corrugata Slade* 939/1 *^T^*	99.35	85.04	31.5
*Pseudomonas chlororaphis subsp. chlororaphis* DSM 50083 *^T^*	99.29	81.15	27.9
*Pseudomonas lini* DLE411J *^T^*	99.29	86.00	26.8
*Pseudomonas taetrolens* NCTC10697 *^T^*	99.20	77.98	23.6
*Pseudomonas uvaldensis* 20TX0172 *^T^*	99.19	84.65	30.9
*Pseudomonas frederiksbergensis* ERDD5:01 *^T^*	99.08	80.84	27.0
*Pseudomonas chlororaphis subsp. aureofaciens* DSM 6698 *^T^*	98.93	81.19	27.1
*Pseudomonas chlororaphis subsp. piscium* DSM 21509 *^T^*	98.93	81.12	26.9
*Pseudomonas arsenicoxydans* CECT 7543 *^T^*	98.93	81.11	26.9
*Pseudomonas versuta L10.10 ^T^*	98.93	77.84	23.5
*Pseudomonas kielensis* MBT-1 *^T^*	98.93	81.26	27.0
*Pseudomonas lundensis* DSM:6252 *^T^*	98.91	77.85	23.4
*Pseudomonas fluorescens* ATCC 13525 *^T^*	98.85	79.28	25.0
*Pseudomonas chlororaphis subsp. aurantiaca* DSM 19603 *^T^*	98.84	81.29	27.2
*Pseudomonas prosekii* LMG 26867 *^T^*	98.84	81.07	26.4
*Pseudomonas migulae* NBRC 103157 *^T^*	98.82	81.23	27.0
*Pseudomonas paraversuta* V4/DAB/S4/2a *^T^*	98.73	78.07	23.7
*Pseudomonas extremaustralis* DSM 17835 = 14–3 *^T^*	98.67	79.71	25.4
*Pseudomonas asgharzadehiana* SWRI132 *^T^*	98.67	79.59	25.6
*Pseudomonas yamanorum* LMG 27247 *^T^*	98.67	79.55	25.5
*Pseudomonas veronii* CIP 104663 *^T^*	98.66	79.73	25.6
*Pseudomonas weihenstephanensis* DSM 29166 *^T^*	98.66	77.58	23.2

The percentage of homology of the sequence of the housekeeping genes *gyrB, rpoB* and *rpoD* in SAICEU11^T^, and *gyrB* and *rpoD* in SAICEU22^T^ was obtained. Likewise, the homology values obtained are included in Tables 6A,B.

**Table 6A:** Similarity of the *gyrB, rpoB*, and *rpoD* genes of the taxa with valid names and SAICEU11^T^.

Gene	Nearby species	Type strain (T)	Identity (%)
*gyrB*	*Bacillus thuringiensis*	ATCC 10792	92.69
	*Bacillus toyonensis*	BCT-7112	92.64
	*Bacillus cereus*	ATCC 14579	91.96
	*Bacillus anthracis*	Vollum strain	91.55
	*Bacillus mycoides*	ATCC 6462	90.93
	*Bacillus mycoides*	DSM 2048	90.62
	*Bacillus cytotoxicus*	NVH 391–98	86.9
*rpoB*	*Bacillus thuringiensis*	ATCC 10792	97.7
	*Bacillus toyonensis*	BCT-7112	97.4
	*Bacillus mycoides*	DSM 2048	97.26
	*Bacillus mycoides*	ATCC 6462	97.15
	*Bacillus cereus*	ATCC 14579	96.79
	*Bacillus anthracis*	Vollum strain	96.71
	*Bacillus cytotoxicus*	NVH 391–98	91.2
*rpoD*	*Bacillus toyonensis*	BCT-7112	94.67
	*Bacillus thuringiensis*	ATCC 10792	93.74
	*Bacillus anthracis*	Vollum strain	93.14
	*Bacillus cereus*	ATCC 14579	92.27
	*Bacillus mycoides*	DSM 2048	91.4
	*Bacillus mycoides*	ATCC 6462	91.2

**Table 6B:** Similarity of the *gyrB* and *rpoD* genes of the taxa with valid names and SAICEU22^T^.

Gene	Nearby species	Type strain (T)	Identity (%)
*rpoD*	*Pseudomonas alvandae*	SWRI17	98.43
	*Pseudomonas viciae*	11 K1	95.94
	*Pseudomonas rhizofila*	S211	95.73
	*Pseudomonas zarinae*	SWRI108	95.67
	*Pseudomonas fluorescens*	F113	95.51
*gyrB*	*Pseudomonas alvandae*	SWRI17	96.94
	*Pseudomonas zarinae*	SWRI108	94.87
	*Pseudomonas fluorescens*	F113	94.75
	*Pseudomonas viciae*	11 K1	94.09
	*Pseudomonas rhizofila*	S211	93.22

### 3.3. Plant growth-promoting characterization

The SAICEU11^T^ strain presented PGP activities such as the production of auxins (concentration of 5.61 ± 5.61 acid 5.61 0.26 μg mL^−1^), production of the enzyme 1-aminocyclopropane-1-carboxylate deaminase (ACCd). The ability to solubilize phosphates was negative. The complete list of genes involved in promoting plant growth is shown in Supplementary Table 2.

The SAICEU22^T^ strain presented the following PGP activities: production of auxins (concentration of acid-3-indoleacetic acid 5.85 ± 0.12 μg mL^−1^), production of ACCd and biosynthesis of siderophores (1.00 ± 0.30 cm). The ability to solubilize phosphates was negative. The complete list of genes involved in promoting plant growth is shown in Supplementary Table 3.

### 3.4. Description and phenotypic and genomic characterization of SAICEU11^T^

#### 3.4.1. Chemotaxonomic features

The cellular fatty acid profiles (> 1%) of strain SAICEU11^T^ and closely related strains are shown in Table 7. The mayor fatty acids (> 10%) of strain SAICEU11^T^ was iso-C_15:0_ (30.96%). We find greater similarity with *Bacillus wiedmannii* FSL W8-0169^T^ whose main fatty acids were iso-C_15:0_ (27.6%) and iso-C_17:0_ (10.1%). The major fatty acids of *Bacillus pacificus* EB422^T^ were C_16:0_ (19.9%) and iso-C_15:0_ (13.8%), same as *Bacillus mobilis* 0711P9-1*^T^* with C_16:0_ (16.7%) and iso-C_15:0_ (10.8%). The SAICEU11^T^ strain can be differentiated from major and minor fatty acids.

**Table 7:** Cellular fatty acid profiles (> 1%) of strain SAICEU11^T^ and type strains of closely related species.

	**1**	**2**	**3**	**4**	**5**
iso-C_12:0_	TR	NR	0.8	1.2	1.9
C_12:0_ 2OH	TR	NR	ND	ND	ND
C_12:0_	ND	NR	TR	1.7	1.8
iso-C_13:0_	5.91	NR	6.9	6.4	4.9
anteiso-C_13:0_	1.37	NR	1.0	2.0	1.8
iso-C_14:0_	4.72	NR	5.1	3.5	5.4
C_14:0_	3.64	NR	3.3	6.8	3.6
iso-C_15:0_	**30.96**	NR	**27.6**	**13.8**	10.8
iso-C_15:1_ G	ND	NR	ND	TR	TR
anteiso C_15:0_	8.25	NR	4.0	6.7	6.5
C_15:0_ 2-OH	ND	NR	TR	ND	ND
C_16:1_ *ω*7*c* alcohol	1.32	NR	1.9	TR	1.7
iso-C_16:0_	7.13	NR	9.1	5.3	9.1
C_16:1_ *ω*11*c*	ND	NR	1.1	1.7	1.3
C_16:0_	4.55	NR	7.3	**19.9**	**16.7**
iso-C_17:1_ *ω*10*c*	4.73	NR	4.7	1.7	2.4
iso-C_17:1_ *ω*5*c*	3.91	NR	2.6	1.0	ND
anteiso C_17:1_ A	1.28	NR	TR	TR	1.0
iso-C_17:0_	7.61	NR	**10.1**	5.2	6.0
anteiso C_17:0_	3.18	NR	1.5	2.7	3.2
anteiso C_17:1_	ND	NR	ND	ND	ND
C_18:1_ *ω*9*c*	ND	NR	ND	3.0	3.0
C_18:0_	TR	NR	TR	2.0	3.4
Sum In Feature 2	2.26	NR	ND	TR	TR
Sum In Feature 3	7.25	NR	ND	8.0	6.6
Sum In Feature 8	ND	NR	ND	4.6	5.6

Strains: 1, SAICEU11^T^; 2, *Bacillus toyonensis* BCT-7112^T^; 3, *Bacillus wiedmannii* FSL W8-0169^T^; 4, *Bacillus pacificus* EB422^T^; 5, *Bacillus mobilis* 0711P9-1*^T^*. NR, no reporter; ND, no detected; TR, trace amount (< 1%). Values are percentages of total fatty acids. Major components (> 10%) are shown in bold.

#### 3.4.2. Phenotypic characterization

The SAICEU11^T^ strain is a Gram-positive bacillus. In nutrient agar, the colonies are observed round (Ø 0. 6 ± 0.2 cm) and a creamy white color. It is catalase positive. In blood agar, the bacteria behave like beta hemolytic. It has motility. The rest of the phenotypic results and biochemical tests are found in Table 8.

**Table 8:** Differential characteristic phenotype of SAICEU11^T^ and closely related *Bacillus* species.

Feature	Units	1	2	3
Cell size	μm	2 ± 0.10 × 0.70 ± 0.15	2.8 × 1.2	2.5–3.2 × 1.2–1.6
Isolation source	−	*Medicago sativa* rhizosphere	Raw milk stored in a silo	Sediment of the Indian Ocean
Temperature range	°C	4–37	5–43	10–39
OGT	°C	28	20–40	30
pH range	−	5.5–8.0	5.0–10.0	5.0–10.0
pH	−	7.0	On	7.0
Substrate	Kind of utilization			
Pyoverdine	Synthesis	−	On	On
Gelatin	Hydrolysis	+	+	−
Arginine	Hydrolysis	−	−	−
Urea	Hydrolysis	−	−	−
Nitrate	Reduction	−	+	On
Phenylalanine	Oxidation	−	On	On
D-glucose	Oxidation	−	+	−
D-glucose	Fermentation	+	+	On
Lactose	Fermentation	+	On	On
D-galactose	Fermentation	−	−	+
L-arabinose	Assimilation	−	−	−
D-Mannitol	Assimilation	+	−	On
Citrate	Assimilation	−	+	+
Sorbitol	Assimilation	−	−	On
Adonitol	Assimilation	−	−	On
Maltose	Assimilation	−	+	On
Xylose	Assimilation	−	−	On

Strains: 1. SAICEU11^T^; 2 *Bacillus wiedmanni* FSL W8-01 69^T^ (Miller et al., 2016); 3. *Bacillus mobilis* MCCC 1 A05 942 (0711P9-1^T^; Liu et al., 2017). Data for the related strains obtained from indicated respective references/database. +, Positive; −, negative; NA, data not available; *M. sativa*: *Medicago sativa*.

The protein profile obtained with the mass spectrometry performed in MALDI-TOF Identifies SAICEU11^T^ within the *Bacillus cereus* Group (Table 9). The spectrum can be consulted in the Supplementary Figure S1.

**Table 9:** Report of matrix-assisted laser desorption/ionization time-of-flight (MALDI-TOF) results.

Sample number	SAICEU11^T^
Type of microorganism	Bacteria
Name of microorganisms	*Bacillus cereus* group
Confidence level (%)	99.9
Confidence level	High

The antibiogram obtained shows that the SAICEU11^T^ strain has low MIC (Supplementary Table 4) compared to the most widely used antibiotics against *Bacillus* spp. This phenotype is compatible with the resistome, which can be consulted at length in the Supplementary Tables 6A,B.

#### 3.4.3. Sequence analysis: ANI and dDDH analysis

The genome size of SAICEU11^T^ was 5,385,342 bp. A total of 90 contigs were found, with the N50 being 170,108 bp in size. The % GC of the strain was 35.30%. The genome was deposited at the NCBI, with the following access numbers (BioProyect PRJNA850797, BioSample SAMN29203362 and GenBank JAOXJG000000000).

The highest dDDH of the SAICEU11^T^ strain was 69.6% versus *Bacillus wiedmanni* FSL W8-01 69^T^. Likewise, the ANI value (mean number of nucleotides) compared between *Bacillus wiedmanni* FSL W8-01 69^T^ and SAICEU11^T^ was 95.99%. Both values were below the recommended cut-off points for the definition of species, set at > 70% (dDDH) and 95–96% (ANI; Baek et al., 2019, Volpiano et al., 2021).

#### 3.4.4. Phylogenetic analysis based on whole genome sequencing (WGS)

**Figure 5:**
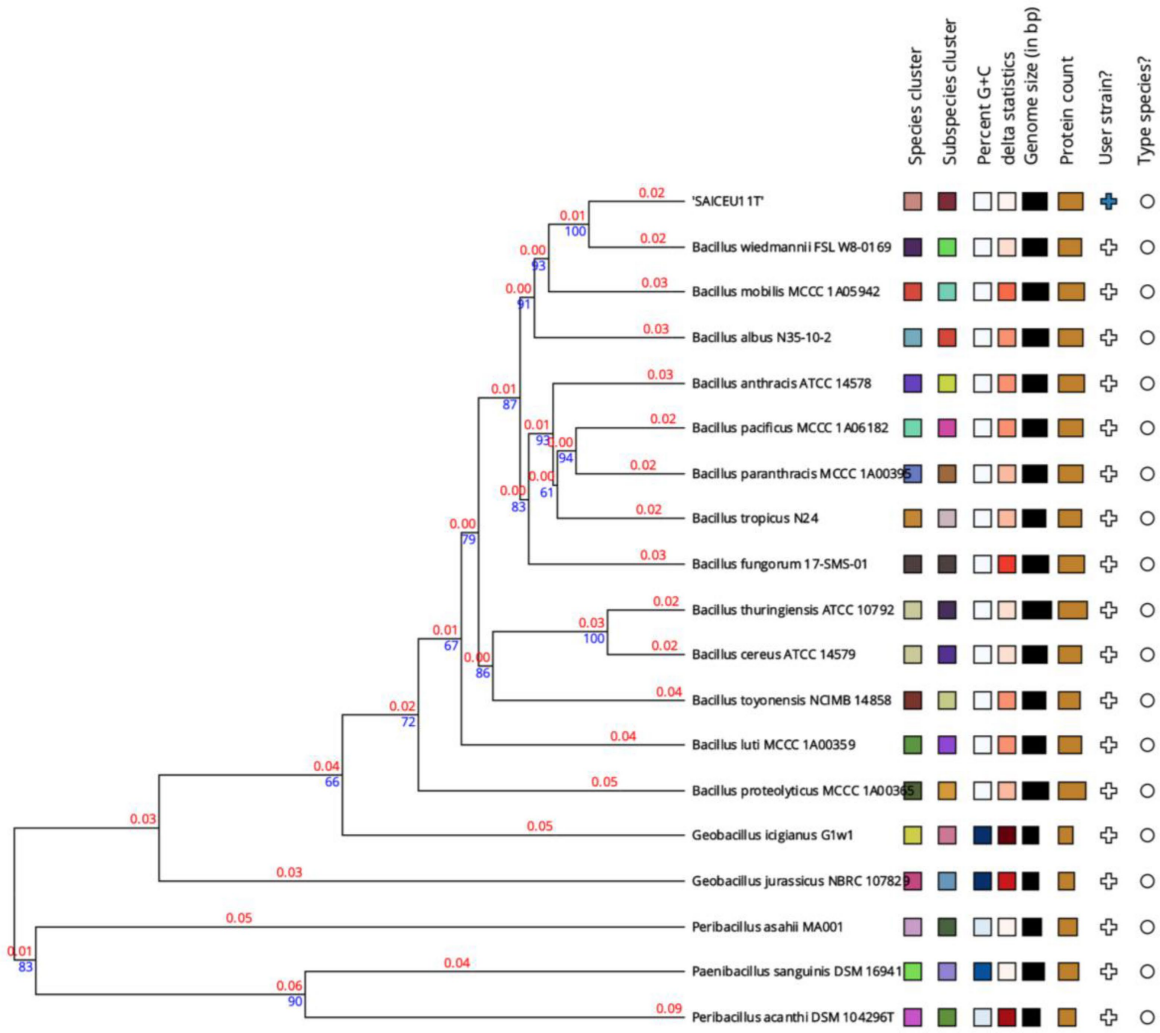
Phylogenetic tree from the complete genome of the species of the genus *Bacillus*. Numerical values indicate the relative distance between the analyzed species. The branch lengths are scaled in terms of GBDP distance d5 formula. The blue numbers are GBDP pseudo-bootstrap support values > 60% from 100 replications, with an average branch support of 55.2%. The branch length values (in red) represent the evolutionary time between two nodes. Unit: substitutions per sequence site. The tree was rooted at the midpoint (Farris, 1972).

A phylogenetic tree was constructed based on the comparison of the complete genome sequences closer to SAICEU11^T^, available in the TYGS database (Figure 5). To do this, the ten strains with the smallest MASH distance were chosen. In the analysis of the phylogenetic tree of the complete genome of the SAICEU11^T^ strain, it is observed that it is in a cluster shared with *Bacillus wiedmannim* and *Bacillus mobilis*.

### 3.5. Description and phenotypic and genomic characterization of SAICEU22^T^

#### 3.5.1. Chemotaxonomic features

The cellular fatty acid profiles (> 1%) of strain SAICEU22^T^ and closely related strains are shown in Table 10. The mayor fatty acids (> 10%) of strain SAICEU22^T^ were C_16:0_ (31.53%), Sum In Feature 3 (25.65%) and Sum In Feature 8 (18.59%). We find greater similarity with *Bacillus wiedmannii* FSL W8-0169^T^ whose main fatty acids were C15:0 iso (27.6%) and iso-C_17:0_ (10.1%). The major fatty acids of *Bacillus pacificus* EB422^T^ were C_16:0_ (19.9%) and iso-C_15:0_ (13.8%), same as *Bacillus mobilis* 0711P9-1*^T^* with C_16:0_ (16.7%) and iso-C_15:0_ (10.8%). No data found for *Pseudomonas alvandae* SWRI17*^T^* and *Pseudomonas rhizophila* S211T*^T^*. In the case of *Pseudomonas canavaninivorans* HB002*^T^* they only refer to the main fatty acids found without percentage, these are C_16:0_, C_16:1_ cyclo *ω*7*c* and C_18:1_ *ω*7*c*. The same happens with *Pseudomonas bijieensis* L22-9 *^T^*, where they describe the main fatty acids, these are C_16:0_, Sum In Feature 3 and Sum In Feature 8. With the current data, more similarity would be found with *Pseudomonas bijieensis* L22-9 *^T^*.

**Table 10:** Cellular fatty acid profiles (> 1%) of strain SAICEU22^T^ and type strains of closely related species.

	**1**	**2**	**3**	**4**	**5**
C_10:0_ 3OH	3.22	NR	NR	NR	NR
C_12:0_ 2OH	4.37	NR	NR	NR	NR
C_12:0_ 3OH	4.18	NR	NR	NR	NR
C_12:0_	2.39	NR	NR	NR	NR
C_14:0_	TR	NR	NR	NR	NR
C_16:1_ cyclo *ω*7*c*	NR	NR	NR	**>10**	NR
C_16:0_	**31.53**	NR	NR	**>10**	**>10**
C_17:0_ cyclo	8.02	NR	NR	NR	NR
C_17:0_	TR	NR	NR	NR	NR
C_18:1_ *ω*7*c*	NR	NR	NR	**>10**	NR
C_18:0_	TR	NR	NR	NR	NR
C19:0 cyclo *ω*8*c*	TR	NR	NR	NR	NR
Sum In Feature 3	**25.65**	NR	NR	NR	**>10**
Sum In Feature 8	**18.59**	NR	NR	NR	**>10**

Strains: 1, SAICEU22^T^; 2, *Pseudomonas alvandae* SWRI17*^T^*; 3, *Pseudomonas rhizophila* S211T*^T^*; 4, *Pseudomonas canavaninivorans* HB002*^T^*; 5, *Pseudomonas bijieensis* L22-9 *^T^*. NR, no reporter; ND, no detected; TR, trace amount (< 1%). Values are percentages of total fatty acids. Major components (> 10%) are shown in bold.

#### 3.5.2. Phenotypic characterization

The SAICEU22^T^ strain is a Gram-negative bacillus. In nutritive agar, the colonies are observed round and whitish (Ø 0.4 ± 0.2 cm). It is catalase positive and has no motility. The rest of the phenotypic results and biochemical tests are found in Table 11.

**Table 11:** Differential characteristic phenotype of *Pseudomonas agronomica* sp. nov. and closely related *Pseudomonas* spp.

Feature	Units	1	2	3
Cell size	(m)μ	1.40 ± 0.10 × 0.50 ± 0.15	1.0–1.5 × 0.5	1.0–1.2 × 2.5–3.3
Isolation source	−	*M. sativa*	*A. thaliana*	Bean
Temperature range	°C	5–40	On	4-37
OGT	°C	28	On	On
pH range	−	5.5–8.0	On	5.5–8.0
OpH	−	7	On	On
*Substrate*	*Kind of utilization*			
Pyoverdine	Synthesis	+	+	+
Gelatin	Hydrolysis	+	+	−
Arginine	Hydrolysis	−	−	On
Urea	Hydrolysis	−	−	−
Nitrate	Reduction	+	+	+
Phenylalanine	Oxidation	−	−	−
D-glucose	Oxidation	+	+	+
D-glucose	Fermentation	−	−	−
Lactose	Fermentation	−	−	−
D-galactose	Fermentation	−	−	−
L-arabinose	Assimilation	−	+	+
D-Mannitol	Assimilation	+	+	+
Citrate	Assimilation	+	+	+
Sorbitol	Assimilation	−	+	On
Adonitol	Assimilation	−	−	On
Maltose	Assimilation	+	−	−
Xylose	Assimilation	+	+	On

Strains: 1. SAICEU22^T^; 2. *Pseudomonas thivervalensis* DSM13194^T^ (Achouak et al., 2000); 3. *Pseudomonas canavaninivorans* HB002^T^ (Hauth et al., 2022). Data for the related strains obtained from indicated respective references/database. +, Positive; −, negative; NA, data not available; *M. sativa*: *Medicago sativa*; *A. thaliana*: *Arabidopsis thaliana*.

The protein profile obtained with the mass spectrometry performed in MALDI-TOF did not correspond to any of the currently known species of the genus *Pseudomonas* (Table 12). The spectrum can be consulted in the Supplementary Figure 1.

**Table 12:** Report of MALDI-TOF results.

Sample number	SAICEU22^T^
Type of microorganism	Bacteria
Name of microorganisms	–
Confidence level (%)	–
Confidence level	Low

The obtained antibiogram shows that the SAICEU22^T^ strain presents low MIC (Supplementary Table 5) compared to the antibiotics of more widespread use against *Pseudomonas* spp. This phenotype is also compatible with the resistome, which can be consulted at length in the Supplementary Tables 7A,B.

#### 3.5.3. Sequence analysis: ANI and dDDH analysis

The genome size of SAICEU22^T^ was 6,158,284 bp. A total of 1,203 contigs were found, with the N50 being a size of 361,685 bp. The % GC of the strain was 61.10%. The genome was deposited at the NCBI, with the following access numbers (BioProject PRJNA853909, BioSample SAMN30035438, GenBank JAOSHO000000000).

For SAICEU22^T^ strain, the highest dDDH rate was 35.40% compared to *Pseudomonas thivervalensis* 21629^T^. Likewise, the ANI value compared between *Pseudomonas thivervalensis* 21629^T^ and SAICEU22^T^ was 87.95%. Both values were below the recommended cut-off points for the definition of species, set at 70% (dDDH) and 95–96 (ANI; Baek et al., 2019; Volpiano et al., 2021).

#### 3.5.4. Phylogenetic analysis based on whole genome sequencing (WGS)

**Figure 6:**
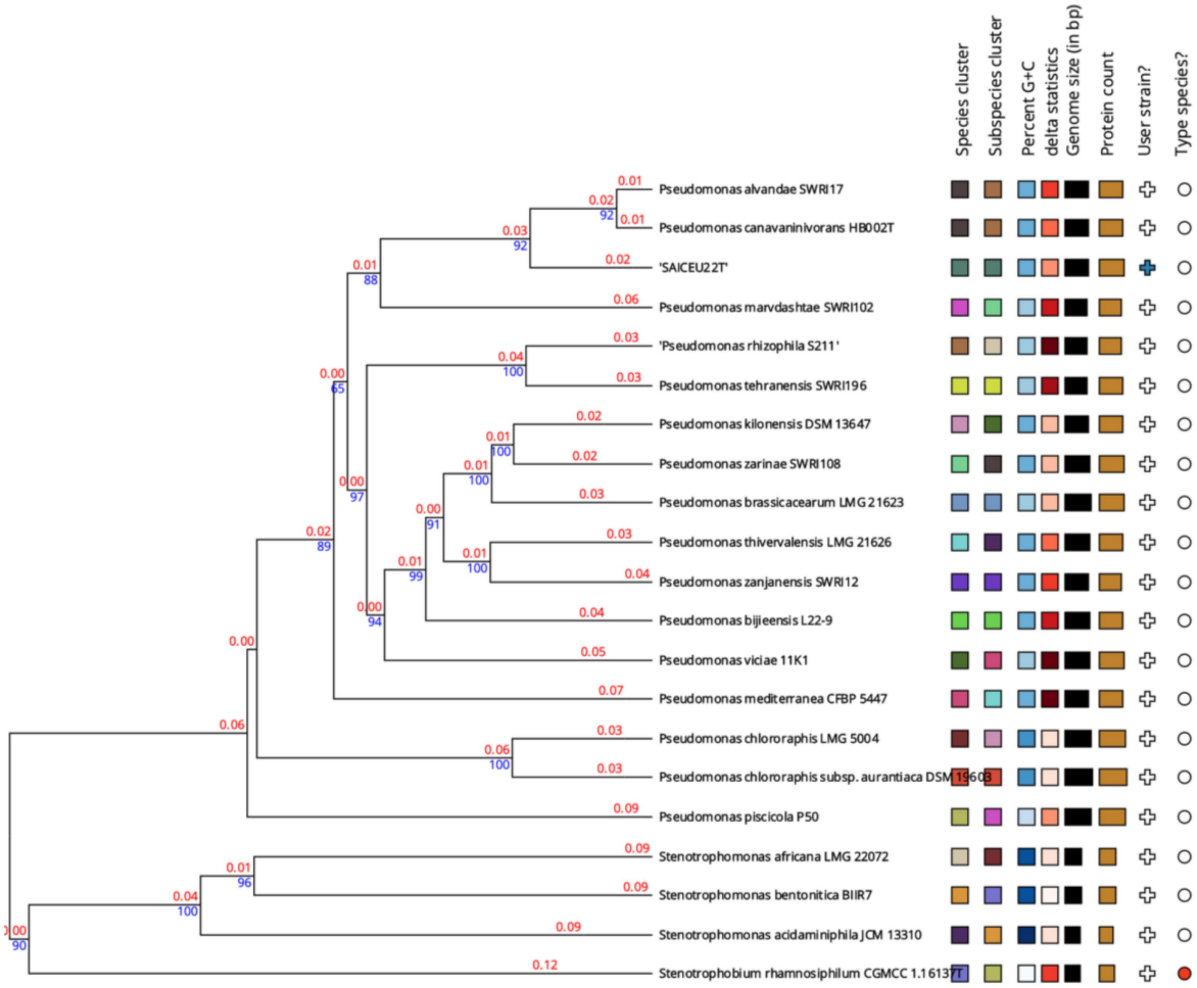
Phylogenetic tree from the complete genome of the species of *Pseudomonas* genus. Numerical values indicate the relative distance between the analyzed species. The branch lengths are scaled in terms of GBDP distance formula d5. The blue numbers are GBDP pseudo-bootstrap support values > 60% from 100 replications, with an average branch support of 55.2%. The branch length values (in red) represent the evolutionary time between two nodes. Unit: substitutions per sequence site. The tree was rooted at the midpoint (Farris, 1972).

A phylogenetic tree was constructed based on the comparison of the complete genome sequences closer to SAICEU22^T^, available in the TYGS database (Figure 6). To do this, the ten strains with the smallest MASH distance were chosen. It is observed that SAICEU22^T^ is segregated from the rest of the species belonging to the *Pseudomonas* genus.

## 4. Discussion

Unlike synthetic chemical fertilizers, organic fertilizers generated from recovered agronomic residues provide a set of nutrients in the form of complex biomolecules, which plants cannot always adsorb directly. However, the addition of PGPB to an organic fertilizer, giving rise to a biofertilizer, induces a double effect. Directly, PGP activities induce the promotion of plant growth. Indirectly, microbial participation in the hydrolysis and transformation of complex organic molecules into simpler nutrients makes them more accessible for root adsorption (Lara Mantilla et al., 2011; Velasco-Jiménez et al., 2020).

There are numerous strains belonging to *Bacillus* and *Pseudomonas* genera that present PGP activities, which allows their potential use to produce biofertilizers (Xue et al., 2021). PGP activities include, among others, the production of auxins (Pereira et al., 2019) and the enzyme ACCd (Kumar et al., 2021; Soumare et al., 2021). The biosynthesis of HCN, encoded by the *hcnABC* operon, induces a phytoprotective effect minimizing the ability to compete with other microorganisms that could behave as phytopathogens. Genetic analysis of SAICEU11^T^ as SAICEU22^T^ shows that both fully possess functional genes for these PGP activities. Additionally, both strains have many other genes that endow them with thermoprotective and phytoprotective capacity (Supplementary Tables 1, 2), not having found genes that encode pathogenicity factors in either of the two studied strains. Thus, the joint and orderly expression of PGP genes can explain their phenotypic profile *in vitro*, as the results of the biometric variables of plants treated with ORGAON^®^ and ORGAON^®^st supplemented with these bacteria.

The comparative fertilization assay of Chem-F added with SAICEU11^T^ to its unadded Chem-F control shows the absence of significant differences in most variables. This fact shows that the addition of the SAICEU11^T^ strain does not induce a better use of nutrients by the plant when tested in a traditional chemical fertilizer. Likewise, there are no significant differences in biometric variables when comparing Chem-F treatments against ORGAON^®^. On the contrary, the use of the biofertilizer resulting from the addition of the SAICEU11^T^ strain, both to ORGAON^®^ and ORGAON^®^st, induces a significant increase in the variables root weight, weight and total length of the plant, and number of secondary roots. This fact shows the double effect that allows a PGPB to contribute to the complex organic fertilizer matrix (Hakim et al., 2021). This indirect activity of nutrient biotransformation has a second positive effect consisting of the microbial retention of biomolecules available to plants (Olanrewaju et al., 2017). In this way, unlike soluble chemical fertilizers, their soil loss by leachate is minimized by remaining longer in the soil and prolonging its availability. The optimization of use by plants can result in the reduction of fertilizer consumption and the minimization of its loss. In this way, the impact on ecosystems is reduced, compared to the use of chemical fertilizers (Stefan et al., 2022).

Treatment with ORGAON^®^st added with the SAICEU22^T^ strain induces a significant increase in both the number of secondary roots and the length of the root. These effects are probably favored by the elimination of competition between the strains tested independently against the microorganisms that originally contained the organic fertilizer ORGAON^®^st prior to its sterilization (Abril et al., 2006). The increase in root size is a very relevant factor, since it allows the plant to acquire nutrients from different depths of the soil, favoring the retention of nutrients that could otherwise be lost by leachate (Olanrewaju et al., 2017).

Regarding the biometric results of the plants treated with the consortium strains, it is observed that there are no significant differences in the treatments with ORGAON^®^, ORGAON^®^st or Chem-F. The fact that the consortium is not effective compared to treatments in which strains are used independently appears to be due to incompatibility or competence phenomena between strains (Kato et al., 2005).

There is a scientific interest in the correct taxonomic ordering and characterization of PGPBs that allows an adequate biotechnological use. Thus, the phenotypic characteristics of the strains SAICEU11^T^ and SAICEU22^T^ place them within the genera *Bacillus* and *Pseudomonas*, *respectively.* The sequence of highly conserved regions and variable regions in the genome of housekeeping *16S rRNA* has allowed for years the identification and classification of bacterial species. Although its use with known clinical strains is very efficient, this application loses reliability when it comes to environmental strains (Gomila et al., 2015). Therefore, although the sequence of a strain present homology of 100% for said *16S rRNA*, the identification with a certain species does not necessarily mean they belong to the same taxon. To verify this, it is necessary to resort to the sequencing of a greater number of housekeeping genes. Currently, the *genes gyrB*, *rpoB* and *rpoD* are widely used both for taxonomic classification and the identification of *Bacillus* genus species (Yamada et al., 1999; La Duc et al., 2004) whilst *gyrB* and *rpoD* are normally used for *Pseudomonas* genus (Yamamoto et al., 2000). The analysis of the homology percentages of the *16S rRNA* gene of the SAICEU11^T^ strain reached a high identity value. However, the analysis of the housekeeping genes *gyrB*, *rpoB* and *rpoD* does not accurately satisfy the criteria for identification of the SAICEU11^T^ strain. In this way, although in the sequence of the *16S rRNA* gene like the rest of housekeeping they present a very high percentage of homology of SAICEU22^T^ against *Pseudomonas alvandae* SWRI17, the identification criteria are not met either (Tables 2, 3). The chemotaxonomic analysis using the protein profile obtained with MALDI-TOF spectrometry, as well as the fatty acid profile do not allow the identification of the strains tested with any known species (Table 2).

The dDDH and ANI analyses show that both species are below the species threshold, which would support the hypothesis that two new species are being treated.

For all these reasons, the SAICEU11^T^ and SAICEU22^T^ strains are defined as not yet described species. Thus, the name of *Bacillus pretiosus* sp. nov is proposed for the strain SAICEU11^T^ (= DSM 114702^T^ = CECT 30674^T^) and *Pseudomonas agronomica* sp. nov for SAICEU22^T^ (= DSM 114959^T^ = CECT 30673^T^) for *Pseudomonas agronomica*.

## 5. Description of *Bacillus pretiosus* sp. nov

*Bacillus pretiosus*, was isolated from the rhizosphere of *Medicago sativa* in the mining district of Almadén (Ciudad Real, Spain), from an abandoned dump of a mercury exploitation. Gram-positive, motile bacillus, ranging from 2 ± 0.10 × 0.70 ± 0.15 μm, catalase positive and with colonies visible at 24 h with a creamy texture and dump color on nutrient agar and with beta-hemolysis on blood agar. Growth occurs between 4 and 37°C in 24 h. It grows in a pH range of 5.5–8.0. It is capable of assimilating D-mannitol, fermenting D-glucose and Lactose, and hydrolyzing gelatin. It can produce auxins and ADDd but does not solubilize phosphates or produce siderophores. The predominant fatty acids for SAICEU11^T^ were C15:0 (39.21%), C16:0 (11.68%), C17:0 (10.79%), C14:0 (8.36%), C13:0 (7.28%) and C17:1 (7.21%). The G + C genomic DNA content of the type strain is 35.30%. The complete genome and the *16S rRNA* gene sequence have been deposited in the NCBI Genbank (BioProject PRJNA850797, BioSample SAMN29203362 and GenBank JAOXJG000000000). The type strain is SAICEU11^T^ (= DSM 114702^T^ = CECT30674^T^).

## 6. Description of *Pseudomonas agronómicas* sp. nov

*Pseudomonas agronomicas* was isolated from the rhizosphere of *Medicago sativa* in the mining district of Almadén (Ciudad Real, Spain), from an abandoned dump of a mercury exploitation. Gram-negative, non-motile bacillus, ranging from 1.40 ± 0.10 × 0.50 ± 0.15 μm, catalase positive, whitish to translucent in color when grown on nutrient agar. In cultures of more than 72 h, it begins to produce pyoverdine, which gives it a characteristic green color. Growth occurs between 5 and 40°C in 24 h and in a pH range of 5.5–8.0. It is capable of assimilating D-mannitol, Citrate, Maltose and Xylose; oxidize D-glucose; reduce Nitrate and hydrolyze Galatin. It produces auxins, ACCd and siderophores but does not solubilize phosphates. The predominant fatty acids for SAICEU22^T^ were C16:0 (31.53%), C12:0 (8.55%), and cyclo C17:0 (8.02%). The G + C genomic DNA content of the type strain is 61.10%. The complete genome and the *16S rRNA* gene of the SAICEU22T strain have been deposited in the NCBI Genbank (BioProject PRJNA850797, BioSample SAMN29203362 and GenBank JAOXJG000000000). The type strain is SAICEU22^T^ (= DSM 114959^T^ = CECT30673^T^).

## Data availability statement

The data presented in the study are deposited in the GenBank repository, accession number JAOSHO000000000 and JAOXJG000000000.

## Author contributions

PJ, VF, and MR: conceptualization. PJ and VF: methodology. VF: software. PJ and MR: validation and supervision. VF and LG: formal analysis and resources. VF, LG, and MR: investigation. PJ, VF, and LG: data curation. LG and MR: writing—original draft preparation. PJ, AP, and MR: writing—review and editing. MR and LG: visualization. AP and PJ: project administration and funding acquisition. All authors contributed to the article and approved the submitted version.

## Funding

This research has been funded by the Fundación Universitaria San Pablo CEU and Banco Santander, grant number FUSP- BS-PPC01/2014. Grants to recognized research groups 2021/2022 (Vice President for Re-search and Teaching Staff, CEU San Pablo University). Likewise, this project has been financed with the European Funds oriented to the ecological transition and digital transition, of the national plan for scientific, technical and innovation research 2021-2023, within the framework of the recovery, transformation and resilience plan. File number TED2021-132285A-I00.

## Conflict of interest

The authors declare that the research was conducted in the absence of any commercial or financial relationships that could be construed as a potential conflict of interest.

## Publisher’s note

All claims expressed in this article are solely those of the authors and do not necessarily represent those of their affiliated organizations, or those of the publisher, the editors and the reviewers. Any product that may be evaluated in this article, or claim that may be made by its manufacturer, is not guaranteed or endorsed by the publisher.
