# Supplementary material for: Plant growth promotion of the forage plant Lupinus albus Var. Orden Dorado using Pseudomonas agronomica sp. nov. and Bacillus pretiosus sp. nov. added over a valorized agricultural biowaste
Source: Front Microbiol. 2023 Jan 26;13:1046201. doi: 10.3389/fmicb.2022.1046201 (PMC9910085; doi:10.3389/fmicb.2022.1046201)

**Supplementary Table 1A.** SAICEU11^T^. Weight biometrics of lupine plants subjected to different irrigation treatments with biofertilizer with SAICEU11T. Different letters denote statistically significant differences according to Duncan test (p < 0.05) for total plant weight (a, ab, and c) and root weight (x, and z).

|  |  |  |  |  |  |  |  |
| --- | --- | --- | --- | --- | --- | --- | --- |
|  | CHEM_F | ORGAON | ORGAON_ST | CHEM_F+SAICEU11 | ORGAON+SAICEU11 | ORGAON_ST+SAICEU11 |  |
| Weight_T (g) | 1.60 ± 0.29^a^ | 1.95 ± 0.55^a,b^ | 1.26 ± 0.33^a^ | 1.57 ± 0.43^a^ | 3.01 ± 0.24^c^ | 2.54 ± 0.39^b,c^ |  |
| Weight_R (g) | 0.21 ± 0.02^x^ | 0.19 ± 0.06^x^ | 0.14 ± 0.03^x^ | 0.17 ± 0.05^x^ | 0.49 ± 0.05^z^ | 0.41 ± 0.06^z^ |  |
|  |  |  |  |  |  |  |  |
| **Supplementary Table 1B.** SAICEU11^T^. Biometrics of lupine plants subjected to different irrigation treatments with biofertilizer with SAICEU11T. Different letters denote statistically significant differences according to Duncan test (p < 0.05) for shoot length (a, b, bc, and c) and the number of secondary roots (x and z). | | | | | | |  |
|  |  |  |  |  |  |  |  |
|  | CHEM_F | ORGAON | ORGAON_ST | CHEM_F+SAICEU11 | ORGAON+SAICEU11 | ORGAON_ST+SAICEU11 |  |
| Weight_T (g) | 1.60 ± 0.29^a^ | 1.95 ± 0.55^a,b^ | 1.26 ± 0.33^a^ | 1.57 ± 0.43^a^ | 3.01 ± 0.24^c^ | 2.54 ± 0.39^b,c^ |  |
| Weight_R (g) | 0.21 ± 0.02^x^ | 0.19 ± 0.06^x^ | 0.14 ± 0.03^x^ | 0.17 ± 0.05^x^ | 0.49 ± 0.05^z^ | 0.41 ± 0.06^z^ |  |
|  |  |  |  |  |  |  |  |
| **Supplementary Table 1C.** SAICEU22^T^. Weight biometrics of lupine plants subjected to different irrigation treatments with biofertilizer with SAICEU22T. Different letters denote statistically significant differences according to Duncan test (p < 0.05) for shoot length (a, ab, abc, bc and c), the root length (o, op, pq and q) and the number of secondary roots (x, y, and z).   \|  \|  \|  \|  \|  \|  \|  \| \| --- \| --- \| --- \| --- \| --- \| --- \| --- \| \|  \| CHEM_F \| ORGAON \| ORGAON_ST \| CHEM_F+SAICEU22 \| ORGAON+SAICEU22 \| ORGAON_ST+SAICEU22 \| \| Length_S (cm) \| 20.23 ± 1.02^b,c^ \| 20.9 ± 1.14^c^ \| 17.29 ± 0.65^a^ \| 20.27 ± 3.04^b,c^ \| 17.58 ± 2.16^a,b^ \| 19.24 ± 1.84^a,b,c^ \| \| Length_R (cm) \| 5.53 ± 0.59^o^ \| 8.17 ± 1.43^o,p^ \| 8.33 ± 0.88^o,p^ \| 8.78 ± 3.38^p,q^ \| 5.52 ± 2.24^o^ \| 12.29 ± 3.55^q^ \| \| Roots (No) \| 21.56 ± 3.06^y^ \| 9.78 ± 1.52^x^ \| 11.17 ± 3.21^x^ \| 13.33 ± 4.49^x^ \| 8.56 ± 4.69^x^ \| 27.93 ± 1.65^z^ \| \|  \|  \|  \|  \|  \|  \|  \| \|  \| \| \| \| \| \| \| | | | | | | |  |

**Supplementary Table 1D.** CS. Weight biometrics of lupine plants subjected to different irrigation treatments with biofertilizer with both SAICEU11^T^ and SAICEU22T (CS). Different letters denote statistically significant differences according to Duncan test (p < 0.05) for the root length (a, ab, and b) and the number of secondary roots (x and y).

|  | CHEM_F | ORGAON | ORGAON_ST | CHEM_F+CS | ORGAON+CS | ORGAON_ST+CS |
| --- | --- | --- | --- | --- | --- | --- |
| Length_R (cm) | 5.53 ± 0.59^a^ | 8.17 ± 1.43^a,b^ | 8.33 ± 0.88^a,b^ | 6.43 ± 2.23^a,b^ | 5.33 ± 2.48^a^ | 8.78 ± 3.40^b^ |
| Roots (No) | 21.56 ± 3.76^y^ | 9.78 ± 1.52^x^ | 11.18 ± 3.21^x^ | 11.43 ± 3.38^x^ | 8.17 ± 6.40^x^ | 13.13 ± 5.17^x^ |

Supplementary Table 2. SAICEU11^T^ strain genes and functionality.

| ACTIVITY | | Regulation | Metabolism/  transformation | Transport | PGP  activity | Reference |
| --- | --- | --- | --- | --- | --- | --- |
| Nitrogen | | *cynR, nirQ, norG* | *narX* | *nirC, narT* |  | Li et al., 2019. |
| Phosphorus | | *PhoP* | *phnD, phnE, pstA, pstB, pstC* & *pstS,* | *phnV* |  | Li et al., 2020. |
| Sulfur | | *cysB.* | *cysL,* | *cysA* |  | Sirko et al., 1995 |
|  |  |  | *tauB*  *sufS, lscS* |  |  | Liang et al., 2021  Bühning et al., 2017 |
| Auxins | |  |  |  | *TsaA,TsaB* |  |
| HCN | |  |  |  | *hcnABC* | Yang et al., 2021 |
| Siderophores | |  | *yusV, yfiY, yfiZ & sbnA*  *entS* |  | *yfhA*  *fepC* | Kang et al., 2020 |
| Environmental  stress | High T |  | *grpE,* |  |  | Kang et al., 2020 |
|  | Low T |  | *cspA, cspD,* |  |  | Liang et al., 2021 |
| Resistance | Hg | *merR* | *merA* |  |  | Boyd & Barkay, 2012 |
|  | Cu | *csoR* | *cutC* | *ycnJ* |  | Kang et al., 2020 |
|  | Mg |  | *corA, corC* |  |  | Kang et al., 2020 |
|  | As |  |  |  |  |  |
| Virulence | |  | *essB, essC, essD (secretion VI)*  *yueB (secretion VII)*  *inhA (inmune inhibitor)*  *capA (capsule)* |  |  | Ates et al., 2016  Guillemet et al., 2010  Ezzell and Welkos, 1999 |
| Motility | |  | *flgB, flgC, flgE, flgF,*  *fliE, fliG, fliN, fliM, fliP, fliY*  *flhA, flhB, flhF* |  |  |  |
| Antibiotic resistance | β-lactam | *BLI* |  |  |  |  |
|  | Fosfomycin | *FosB* |  |  |  |  |
|  | Streptomycin | *SatA* |  |  |  |  |
|  | Ejection bombs | *MATE* superfamily, *AcrB* |  |  |  | Nimmy et al., 2022 |

Supplementary Table 3. SAICEU22^T^ strain genes and functionality.

| ACTIVITY | | Regulation | Metabolism/  transformation | Transport | PGP  activity | Reference |
| --- | --- | --- | --- | --- | --- | --- |
| Nitrogen | | *cynR, nirQ, narL, narX, norR* | *vnfA* | *nrtA, narK* |  | Li et al. 2019. |
| Phosphorus | |  | *phnD, phnE, pstA, pstB, pstC* & *pstS,* | *pqqB, pqqD,* |  | Li et al. 2020. |
| Sulfur | | *cysB.* | *cysA, cysT, cysW* |  |  | Sirko et al., 1995 |
|  |  |  | *tauABCD operon,* |  |  | Liang et al. 2021 |
| ACCd | |  | *accD* |  |  | Glick et al. 2005 |
| Auxins | |  |  |  | *anPRT* |  |
| HCN | |  |  |  | *hcnABC* | Yang et al. 2021 |
| Siderophores | |  |  |  | *pvdQ* |  |
| Environmental  stress | High T |  | *grpE,* |  |  | Kang et al. 2020 |
|  | Low T |  | *cspA, cspD,* |  |  | Liang et al. 2021 |
| Resistance | Hg | *merR* | *merE* | *merC* |  | Boyd & Barkay 2012 |
|  | Cu |  | *copA, copB, copC, copD* | *CopC* |  | Kang et al. 2020 |
|  | Mg |  | *corA, corC* & *mgtE* |  |  | Kang et al. 2020 |
|  | As |  | *arsC* | *arsH* |  |  |
| Virulence | | *pilY1,* | *pilQ (fibria IV)* |  |  | Martin et al. 1993 |
|  |  |  | *vgrG (secretionIV)* |  |  | Boak et al. 2022 |
| Motility | |  | *operon flgBCDEFGJ,*  *operon fliEGHJNMPS,*  *flhA, flhB,* |  |  |  |
| Biofilm | | *mucB*  *pgaD* | *algA, algB, algE, algJ, algK* & *algX* |  |  | ITOH et al. 2008) |
| Antibiotic resistance | β-lactam | *AmpC* |  |  |  |  |
|  | Fosfomycin | *pit* |  |  |  |  |
|  | Streptomycin | *Sata* |  |  |  |  |
|  | Ejection bombs | *macA*  *MATE* superfamily |  |  |  | Nimmy et al. 2022 |

**Supplementary Table 4.** MIC (μg.mL^-1^) of SAICEU11^T^, quantified by the agar diffusion method with E-test strips.

|  | Amoxicilin | Amoxicilin/ Clavulonic acid | Cefepime | Cefotaxime | Cefpirome | Nalidixic acid | Ciprofloxacin | Sulfamethoxazole and trimethoprim | |
| --- | --- | --- | --- | --- | --- | --- | --- | --- | --- |
| MIC (μg.mL^-1^) | 16 | 8 | 16 | 16 | 8 | 2 | 0.125 | 40 |  |

**Supplementary Table 5.** MIC (μg.mL^-1^) of SAICEU22^T^, quantified by the agar diffusion method with E-test strips.

|  | Piperacillin | Piperacillin/ Tazobactam | Ceftazidime | Cefepime | Imipenem | Amikacin | Gentamicin | Ciprofloxacin |
| --- | --- | --- | --- | --- | --- | --- | --- | --- |
| MIC (μg.mL^-1^) | 2 | 2 | 1 | 0.5 | 1 | 0.25 | 0.125 | 0.016 |

**Supplementary Table 6A.** Blastx analysis with the proteins deduced from the genome against the databases of Comprehensive Antibiotic Resistance Database (CARD), ResFinder y ARG-ANNOT V4.

| STRAIN | GENE | GENE_ID | IDENTITY (%) | QUERY | PHENOTYPE |
| --- | --- | --- | --- | --- | --- |
| SAICEU11^T^ | *Bcll* | WP_000742464.1 | 99.60 | 95 | *Bcll* family subclass B1 metallo-beta-lactamase [*Bacillus*] |
|  | *FosB/FosD* | WP_088080719.1 | 99.28 | 99 | *FosB/FosD* family Fosfomycin resistance bacillithiol transferase [*Bacillus*] |
|  | *M15* | WP_000747666.1 | 99.59 | 99 | *M15* family metallopeptidase [*Bacillus wiedmannii*] |
|  | *SMR* | WP_064774856.1 | 96.70 | 76 | Multidrug efflux *SMR* transporter [*Bacillus*] |
|  | *VanW* | WP_046954611.1 | 99.34 | 99 | *VanW* family protein [*Bacillus*] |
|  | *VanA* | WP_264462391.1 | 99.57 | 99 | *VanA-type* vancomycin resistance DNA-binding response regulator *VanR* [*Bacillus*] |
|  | *M15* | WP_000720206.1 | 100 | 99 | *M15* family metallopeptidase [*Bacillus cereus group*] |
|  | *TetO* | WP_264462179.1 | 94.90 | 99 | Tetracycline resistance ribosomal protection protein [*Bacillus thuringiensis*] |
|  | *Beta-lactamase 1* | OUB53290.1 | 996.1 | 99 | Class A beta-lactamase [*Bacillus thuringiensis serovar sylvestriensis*] |
|  | *SMR* | WP_000263259.1 | 100 | 99 | Multidrug efflux *SMR* transporter [*Bacillus*] |
|  | *M15* | WP_071757556 | 97.55 | 99 | *M15* family metallopeptidase [*Bacillus cereus group*] |

**Supplementary Table 6B.** Resistance genes from the annotation of genes of the server of Rapid Annotations using Subsystems Technology

| STRAIN | GENE | SUBSYSTEM | PHENOTYPE |
| --- | --- | --- | --- |
| SAICEU11^T^ | *A, B, Reg* | Multidurg Resistance | A - Multidrug resistance protein.  B – Membrane component of multidrug resistance system.  Reg – *TetR* family regulatory protein of MDR cluster. |
|  | *SatA* | Streptothricin resistance | Streptothricin acetyltransferase, Streptomyces lavendulae type |
|  | *gyrA* | Resistance to fluoroquinolones | DNA gyrase subunit A |
|  | *EF-G, Tet-like* | Tetracycline resistance | EF-G – Translation elongation factor G  Tet-like – Ribosome protection-type tetracycline resistance related proteins |
|  | *FosB* | Fosfomycin resistance | Fosfomycin resistance protein *FosB* |
|  | *gyrA* | Resistance to fluoroquinolones (QRDR mutation) | DNA gyrase subunit A |
|  | *gyrB* | Resistance to fluoroquinolones | DNA gyrase subunit B |
|  | *BLI* | Beta-lactamase | Metal-dependent hydrolases of the beta-lactamase superfamily I |
|  | *AcrB* | Multidrug Resistance Efflux Pumps | Acriflavin resistance protein |
|  | *MDR* | MATE | Multi antimicrobial extrusion protein (Na(+)/drug antiporter), MATE family of MDR efflux pumps |

**Supplementary Table 7A.** Blastx analysis with the proteins deduced from the genome against the databases of Comprehensive Antibiotic Resistance Database (CARD), ResFinder y ARG-ANNOT V4.

| STRAIN | GENE | GENE_ID | IDENTITY (%) | QUERY | PHENOTYPE |
| --- | --- | --- | --- | --- | --- |
| SAICEU22^T^ | *MexB* | WP_030141618.1 | 99.52 | 99 | Multidrug efflux RND transporter permease subunit [*Pseudomonas fluorescens*] |
|  | *BepE* | WP_181290094.1 | 99.90 | 98 | Efflux RND transporter permease subunit [*Pseudomonas brassicacearum*] |
|  | *SoxR* | WP_030137814.1 | 93.38 | 99 | Redox-sensitive transcriptional activator *SoxR* [*Pseudomonas*] |
|  | *EmhB* | WP_181286582.1 | 99.43 | 99 | Efflux RND transporter permease subunit EmhB [*Pseudomonas brassicacearum*]. |
|  | *MFS* | WP_181290492.1 | 99.55 | 99 | MFS transporter [*Pseudomonas*] |

**Supplementary Table 7B.** Resistance genes from the annotation of genes of the server of Rapid Annotations using Subsystems Technology

| STRAIN | GENE | SUBSYSTEM | PHENOTYPE |
| --- | --- | --- | --- |
| SAICEU22^T^ | *BLI* | Beta-lactamase | Metal-dependent hydrolases of the beta-lactamase superfamily I |
|  | *SatA* | Streptothricin resistance | Streptothricin acetyltransferase, Streptomyces lavendulae type |
|  | *BLI* | Beta-lactamase | Metal-dependent hydrolases of the beta-lactamase superfamily I |
|  | *fosA* | Fosfomycin resistance | Fosfomycin resistance protein *FosA* |
|  | *YdhE/NorM* | MATE | Multidrug and toxin extrusión (MATE) family efflux pump *YdhE/NorM*, homolog. |
|  | *MDR* | MATE | Multi antimicrobial extrusion protein (Na(+)/drug antiporter), MATE family of MDR efflux pumps |

**Supplementary Figure 1.** MALDI-TOFF. Mass spectrum of strain SAICEU11^T^ obtained by using the VITEK MS automated mass spectrometry system (bioMérieux). The horizontal axis shows mass/charge ratio (Da) and the vertical axis shows the relative intensities of ions (%). Peaks >12,500 Da are not shown due to their low intensity.


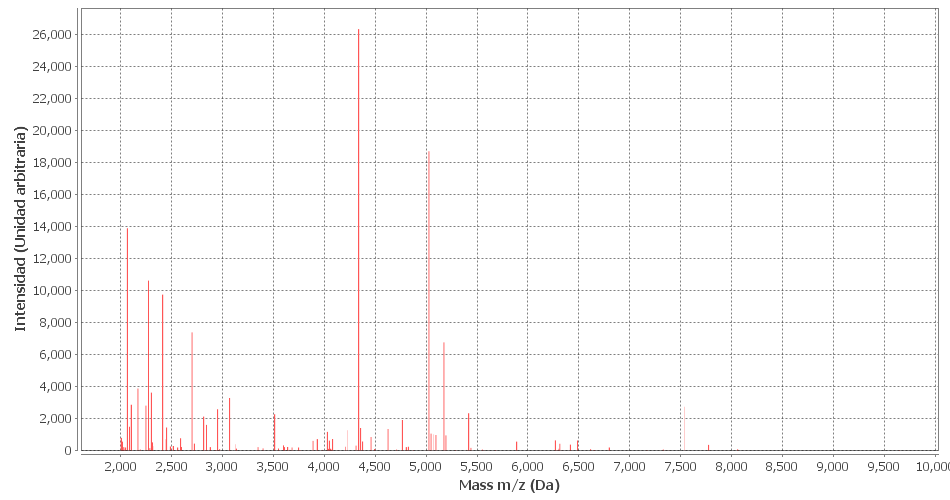


**Supplementary Figure 2.** MALDI-TOFF. Mass spectrum of strain SAICEU22^T^ obtained by using the VITEK MS automated mass spectrometry system (bioMérieux). The horizontal axis shows mass/charge ratio (Da) and the vertical axis shows the relative intensities of ions (%). Peaks >12,500 Da are not shown due to their low intensity.


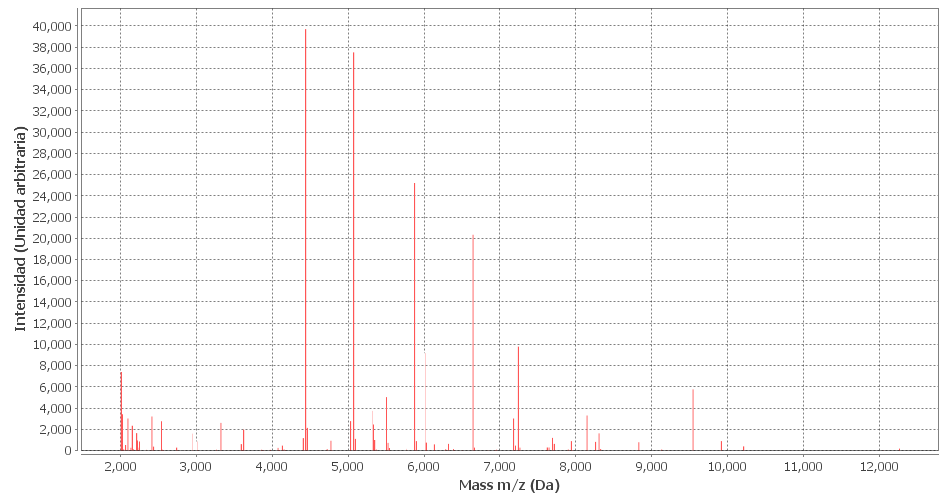

Supplement: Supplementary file 1 [file Data_Sheet_1.docx]
